# Tunable vacuum-field control of fractional and integer quantum Hall phases

**DOI:** 10.1038/s41586-025-08894-3

**Published:** 2025-05-14

**Authors:** Josefine Enkner, Lorenzo Graziotto, Dalin Boriçi, Felice Appugliese, Christian Reichl, Giacomo Scalari, Nicolas Regnault, Werner Wegscheider, Cristiano Ciuti, Jérôme Faist

**Affiliations:** 1 Institute for Quantum Electronics, ETH Zürich, Zürich, Switzerland; 2 Quantum Center, ETH Zürich, Zürich, Switzerland; 3 Université Paris Cité, CNRS, Matériaux et Phénomènes Quantiques, Paris, France; 4 Laboratory for Solid State Physics, ETH Zürich, Zürich, Switzerland; 5 Center for Computational Quantum Physics, Flatiron Institute, New York, NY USA; 6 Laboratoire de Physique de l’Ecole Normale Supérieure, ENS, Université PSL, CNRS, Sorbonne Université, Paris, France; 7 Department of Physics, Princeton University, Princeton, NJ USA

**Keywords:** Quantum Hall, Quantum optics

## Abstract

In quantum mechanics, empty space is not void but is characterized by vacuum-field fluctuations, which underlie phenomena such as the Lamb shift^1^, spontaneous emission, and the Casimir effect^2^. Due to their quantitatively small relative contributions in free-space atomic physics, they were traditionally overlooked in solid-state systems. Recently, however, the interplay between electronic correlations and quantum electrodynamical effects in low-dimensional systems has become a rapidly advancing area in condensed matter physics^3–5^, with substantial implications for quantum materials and device engineering. High-mobility two-dimensional electron gases in the quantum Hall regime^6^ offer an ideal platform to investigate how vacuum electromagnetic fields affect strongly correlated electronic states. Here we demonstrate that adjusting the coupling strength between a two-dimensional electron gas and the vacuum fields of a hovering split-ring resonator leads to a significant reduction in exchange splitting at odd-integer filling factors, along with an enhancement of fractional quantum Hall gaps at filling factors 4/3, 5/3 and 7/5. Theoretical analysis indicates that these effects stem from an effective long-range attractive interaction mediated by virtual cavity photons in regions with strong vacuum electric field gradients. Our findings uncover a new mechanism by which cavity vacuum fields can reshape electronic correlations in quantum Hall systems, establishing a new approach for manipulating correlated quantum phases in low-dimensional materials and paving the way for engineering tailored many-body interactions in compact devices.

## Abstract (web summary)

Using a system to adjust the strength of cavity vacuum fields penetrating a Hall bar, a study describes the effect of the vacuum field of a cavity on electronic correlations in quantum Hall systems.

## Main

In the newly emerged field of cavitronics^5^, research has been leveraging the ultrastrong light–matter coupling regime^7^, aiming to modify molecular structures^8^, enhance electron–phonon couplings^9^ and facilitate the emergence of new electronic phases, including superconductivity^10^, ferroelectricity^11^ and topological properties^12,13^. Despite substantial theoretical backing and evidence of cavity-induced alterations in chemical reactions^3,14^ and charge transport^13,15–19^, demonstration of the manipulation of strongly correlated phases^20^ by cavity vacuum fields remains scarce.

**Fig. 1:**
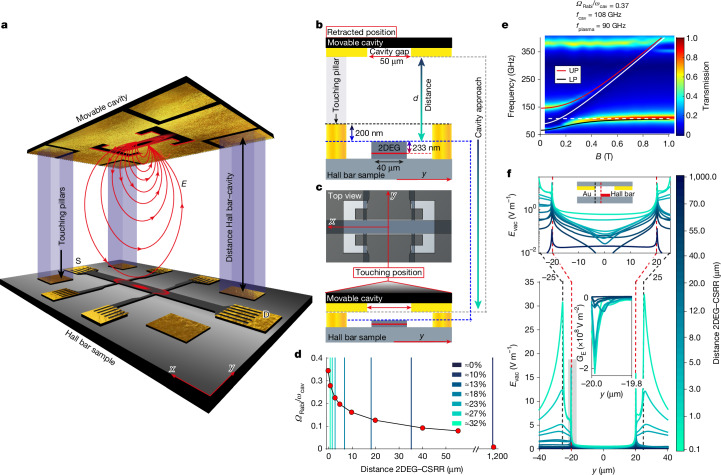
Description of the experimental set-up consisting of a movable split-ring resonator hovering over a Hall bar. **a**, Artistic rendering of the platform implemented to tune the coupling strength between the electrons of the 2DEG within a Hall bar and the vacuum electromagnetic fields of a CSRR. The CSRR, defined by a cutout in a gold layer, approaches the Hall bar from above, increasing the coupling to its fringing electric field, *E* (red arrows). S, source; D, drain. **b**, Side profile along the *y* axis: the resonator plane, with a 50-μm cavity gap (red), is positioned above the Hall bar, which has a width of 40 μm and gold-coloured pillars protruding next to it. The resonator plane is shown in a retracted position, not in contact with the sample, with a separation *d* from the Hall bar surface. **c**, As the cavity moves closer, the resonator plane touches the pillars, establishing electrical contact. At the top, an overlaid view of the Hall bar and CSRR is displayed, with the grey shading representing the CSRR metal, showing their alignment (Methods). **d**, Simulated normalized coupling strength as a function of the distance between the resonator and the 2DEG. The coloured vertical lines correspond to the distances *d* + 233 nm (the quantum well is located at a depth of 233 nm below the surface) at which the magnetotransport traces in Figs. 2a and 3a were measured in the experiment. **e**, Simulated polariton dispersion of the coupled system with the cavity being 0.1 μm away from the 2DEG. UP, upper polariton; LP, lower polariton. **f**, Simulation of the cavity vacuum electric field profile across the Hall bar at magnetic field *B* = 0.3 T. The black dashed line indicates the gap region of the CSRR, and the red dashed line marks the edge of the 2DEG. Top: zoom-in of the area around the Hall bar. Inset bottom: gradient of the vacuum electric field exponentially decaying into the 2DEG (shaded area).

In this work, as illustrated in Fig. 1, we have developed a mobile cavity system capable of finely tuning the strength of cavity vacuum fields permeating a small Hall bar, driving the system in situ from a completely uncoupled state to an ultrastrongly coupled one, hence allowing a fully self-referenced assessment of the impact of vacuum fluctuations on the material system. This is achieved by adjusting the distance between a metallic split-ring resonator and the high-mobility GaAs-based two-dimensional electron gas (2DEG) within a quantum well heterostructure. When subjected to a strong perpendicular magnetic field, this set-up allows for the probing of the integer quantum Hall effect and of strongly correlated fractional quantum Hall (FQH) phases by means of transport measurements of longitudinal and transverse resistance. We have discovered that the ‘hovering’ cavity resonator decreases the exchange splitting at odd filling factors while significantly enhancing several fractional quantum Hall phases with respect to their value in the uncoupled Hall bar. This enhancement occurs while maintaining the metallic resonator at distances for which electrostatic screening effects are entirely negligible. We leverage activated-transport measurements of both integer quantum Hall and FQH gaps as exemplary cases to demonstrate the influence of cavity vacuum fields on condensed matter systems, and how to exploit them to address in situ quantum states in small devices.

This experiment demonstrates that quantum light–matter interaction can reduce spin splittings and enhance certain fractional quantum Hall gaps, and our theoretical analysis shows that this effect can arise from a cavity-mediated attractive potential. Further investigations using complementary techniques, such as capacitance measurements^21–24^ and inelastic light scattering^25–28^, which have been used to study fractional quantum Hall states at filling factors *ν* < 1, will be essential for achieving a comprehensive understanding of these effects.

**Extended Data Fig. 1:**
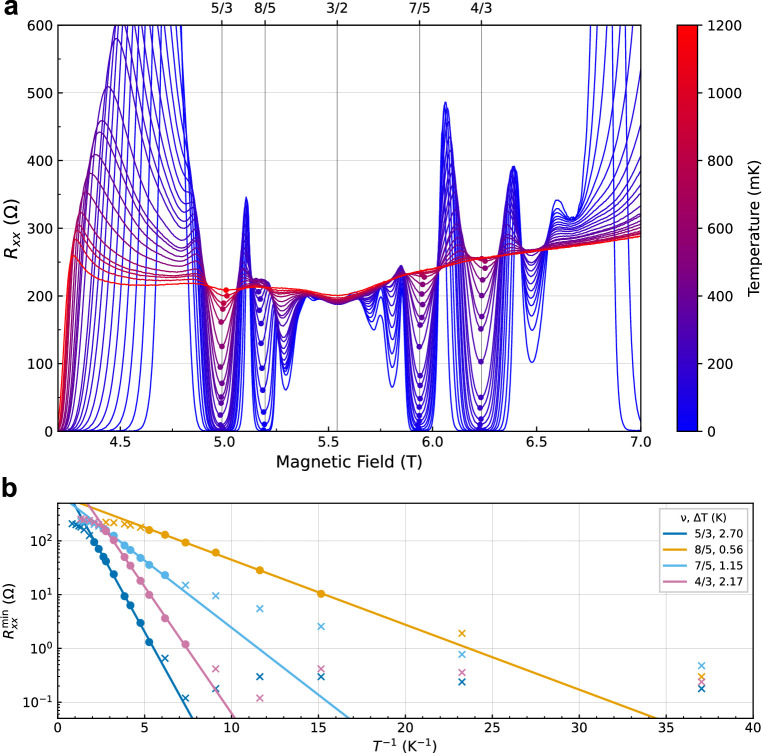
Measurement of FQH gaps in van der Pauw’s geometry. **a**: Longitudinal resistance as a function of magnetic field between filling factors 1 and 2, as measured with van der Pauw’s method on the same heterostructure D151202B employed for the samples presented in the main text. Data measured at different temperatures are displayed with different colors, according to the colorbar on the right. The fractional filling factors from which the energy gap is investigated are indicated on the top axis. Minima of the resistance at these fillings are indicated with circle markers, with color according to the temperature. **b**: Minima of the longitudinal resistance (in log scale) as a function of inverse temperature, as obtained from the circle markers in **a**, for the different fractional fillings (indicated in the legend). The data are fitted to the Arrhenius law ${R}_{xx}^{\min }\propto \exp (-\Delta T\,/2T)$, which allows to retrieve the fractional gap Δ*T*, reported in K in the legend, next to the corresponding fractional filling. Circle markers indicate the data points which have been employed in the fitting procedure, since they display a linear dependence (in log scale), while data excluded from the fitting are indicated with cross markers.

**Extended Data Fig. 2:**
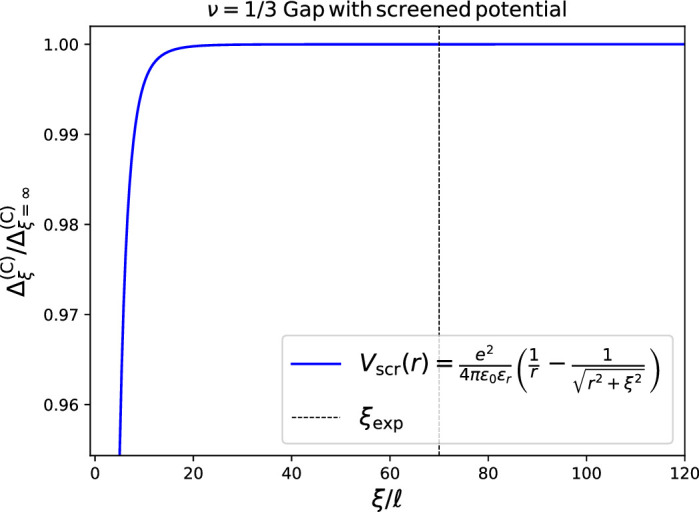
Evolution of the 1/3 fractional quantum Hall energy gap with a Coulomb potential screened by a metal plane at a distance *ξ*/2. The gap is normalized to the same quantity calculated for the bare Coulomb potential. The distance is expressed in units of the magnetic length *ℓ*. The result has been obtained by exact diagonalization with *N* = 5 electrons. In the experiments *ξ* > 2*d* = 700 nm (due to the GaAs buffer on top of the quantum well). For *B* = 5 T, the magnetic length *ℓ* ≃ 10 nm. Hence, for our experimental set-up, we have *ξ*/*ℓ* > 70. As shown by the figure, the variation of the fractional quantum Hall gaps is totally negligible at such distances. Note also the tiny decrease of the gap at much shorter distances.

Our primary focus is to highlight the impact of vacuum fields on solid-state systems, using integer and fractional quantum Hall effects as key examples. In this context, it is important to note that the energy gaps of FQH states are highly sensitive to system parameters. When measured through activated transport, the activation energy extracted by means of Arrhenius law fitting corresponds to the energy required to create a widely separated composite-fermion quasiparticle–quasihole pair^29^. This activation energy—corresponding to the energy gap—can vary across different systems, even for the same material. It depends on factors such as electron mobility^30–32^, effective layer thickness^33^, local disorder^34,35^ and Hall bar geometry, as demonstrated with the reference measurements on the same heterostructure in van der Pauw geometry reported in Extended Data Fig. 1 (see the Supplementary Information for details on analysis and measurement). Additionally, these gaps strongly depend on the nature of electron–electron interactions. Previous theoretical research^36^ has highlighted the potential to enhance these gaps in monolayer two-dimensional (2D) materials such as graphene by leveraging the electrostatic screening effects of a closely positioned dielectric layer, of the order of the electron’s magnetic length. However, such a strategy is generally infeasible for GaAs quantum wells, which are embedded within a thicker semiconductor layer with a high dielectric constant that prevents close-proximity adjustments. Furthermore, the substitution of a dielectric layer with a metallic one is expected to reduce the gap^36^. Indeed, we have independently recalculated the fractional quantum Hall gaps taking into account the Coulomb potential modified by the image charges produced by a metallic plate and found agreement with ref. ^36^ for their case *α* = −1. In this case, there is a reduction of the Laughlin gap. Moreover, when the distance is much larger than the magnetic length, as in our experiments, the magnitude of the change is totally negligible (Extended Data Fig. 2). Additional details are provided in the Supplementary Information.

In our experiment, we utilize a metallic resonator positioned at a distance from the 2DEG, typically orders of magnitude larger than the magnetic length, a range at which electrostatic modifications to the Coulomb potential become negligible. Moreover, contrary to expectations of electrostatics in the presence of a metal plate, the metallic resonator does not decrease but rather increases the fractional quantum Hall gaps. We interpret this remarkable effect as stemming from the emergence of a cavity-induced long-range attractive electron–electron potential mediated by the exchange of virtual cavity photons. In particular, the presence of strong spatial gradients of the vacuum fields is essential to create a cavity-mediated electron–electron interaction within the same Landau level, which is, in the absence of those gradients, forbidden by Kohn’s theorem^19^.

The experimental set-up depicted in Fig. 1a is designed to vary the spacing between a Hall bar and a cavity, specifically a complementary split-ring resonator (CSRR) evaporated on a GaAs substrate. Adjusting this distance allows us to modulate the intensity of the cavity’s fringing fields, which extend beyond the resonator’s gap (illustrated by red lines). These fields penetrate the Hall bar and interact with the electron gas. Figure 1c shows the alignment of the CSRR’s 50-μm gap with the 40-μm-wide Hall bar, which was crafted using conventional photolithography on a GaAs-based heterostructure. The quantum well beneath the surface, located at a depth of 233 nm, exhibits a high electron mobility of 1.69 × 10^7^ cm^2^ V^−1^ s^−1^ and a sheet density of 2.06 × 10^11^ cm^−2^ at 1.3 K without illumination.

Adjacent to the Hall bar are four etched pillars rising 200 nm above the surface, effectively spacing the CSRR’s gold plane from the Hall bar to prevent physical contact and serving as alignment references for the initial set-up. Finite-element simulations, detailed in Fig. 1d, explore the coupling strength *Ω*_Rabi_/*ω*_cav_ as a function of distance—in which *Ω*_Rabi_ represents the Rabi frequency^37^, and *ω*_cav_ = 2π*f*_cav_ denotes the cavity’s angular frequency. By fitting the Hopfield model’s anti-crossing curve to the polaritonic dispersion (Fig. 1e), it is demonstrated that the system can be finely adjusted from a nearly decoupled state (*Ω*_Rabi_/*ω*_cav_ ≈ 0 at a 1,200-μm cavity distance) to an ultrastrong coupling regime^38^ (*Ω*_Rabi_/*ω*_cav_ = 37% as the cavity approaches within 0.1 μm of the 2DEG). As the resonator approaches, the cavity’s resonance frequency shifts downward from 145 GHz to 115 GHz, a change attributed to the fact that the mode field reaches the semiconductor region. Note that measurements were taken at a minimal CSRR-to-Hall bar surface distance of *d* = 0.35 μm, achieving a coupling strength of 32%, in which *d* indicates the separation between the CSRR and the Hall bar surface, ensuring that the resonator plane and the Hall bar sample remain spatially separate (Fig. 1b). By contrast, the simulations pertain to the CSRR’s distance from the 2DEG, which is effectively increased by an additional 233-nm semiconductor layer atop the 2DEG.

In Fig. 1f, we study the vacuum electric field (*E*_vac_) profile across the Hall bar at a magnetic field *B* = 0.3 T for different distances *d*, focusing on the lower polariton branch, while noting that the upper polariton branch shows very similar results as shown in the Supplementary Information.

As the resonator plane is moved closer to the Hall bar, the amplitude of the electric field gradually increases. Around the Hall bar, centred at zero and spanning 40 μm, four symmetric maxima emerge at the 2DEG level. Peaks at ±25 μm align with the CSRR gap edges, and maxima at ±20 μm correspond to the Hall bar edges, where the CSRR’s vacuum electric field *E*_vac_ interacts with the 2DEG’s electrons and then rapidly decays into the bulk, leading to a maximum field gradient of the order of 10^8^ V m^−2^ (Fig. 1f, inset bottom) and to a remaining electric field of the order of 0.8 V m^−1^ within the bulk of the Hall bar when the system is 37% coupled.

During a single cool-down in a dilution refrigerator, we measure the longitudinal resistivity *ρ*_*xx*_ on the side of the Hall bar and the transverse resistance *R*_H_ across the Hall bar as a function of the magnetic field. Each trace is taken for a specified distance *d* and can be associated with the corresponding coupling according to the simulations in Fig. 1d. Measurements are conducted at an electronic temperature of ≈20–45 mK, very close to the mixing chamber temperature. Slight temperature variations below 50 mK due to eddy currents during magnetic field ramping are consistent across all measurements, and the experimentally measured temperature is always considered in the analysis of activation gaps.

**Fig. 2:**
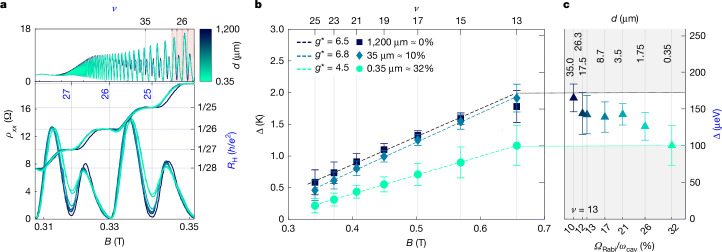
In situ modification of quantum Hall transport and cavity-induced reduction of the exchange splitting and effective *g*-factor. **a**, Longitudinal resistance (left vertical axis) and Hall resistance (right vertical axis) for different distances *d*. As the coupling increases, at odd integer values of the filling factor *ν*, the longitudinal resistance at the minima rises and the correspondent quantum Hall plateaux lose quantization. **b**, Plot of the activation temperatures (left axis)—equivalently, energies (right axis)—extracted from the Arrhenius plot (Supplementary Information) for odd filling factors *ν* = 25 to *ν* = 13. The effective *g*-factor is obtained from a linear fit indicated with dashed lines and reported in the legend together with *d* and the normalized coupling strength. **c**, Evolution of the activation energy of *ν* = 13 as a function of the coupling and corresponding distance *d*. The error bars in **b**,**c** correspond to the standard deviation of the Arrhenius law fit (not weighted), and therefore describe the goodness of the fit.

As illustrated in Fig. 2a (top), at lower magnetic fields and as the resonator plane is gradually brought closer to the Hall bar, increasing the coupling strength, we observe a consistent increase in the minima of the longitudinal resistivity and a gradual degradation of the quantized plateaux (Fig. 2a, bottom). The data suggest that for odd quantum Hall plateaux, the modification of the electron–electron potential by the cavity has the effect of weakening the quantization, as was already observed in a more marked fashion in our previous experiments^18^ in which the cavity was directly fabricated onto the Hall bar (see also the Supplementary Information for further comparison between the two results). We remark that, as recently reported^39^, at magnetic fields at which quantization is present, we do not see evidence for a renormalization of the von Klitzing constant *R*_K_ of either integer or fractional plateaux.

This behaviour at odd filling factors suggests a reduction in effective spin splitting with increased cavity coupling. Indeed, the effective spin splitting Δ is experimentally obtained by studying the thermally activated minima of the longitudinal resistance, which exhibit a characteristic exponential decay as a function of the inverse temperature (see the Arrhenius plots in the Supplementary Information). As shown in Fig. 2b, we observe a linear dependence of the gap Δ as a function of the magnetic field whose slope *μ*_B_*g*^⋆^/*k*_B_ (in which *k*_B_ is Boltzmann’s constant and *μ*_B_ is the Bohr magneton) directly yields^40^ the effective gyromagnetic factor of the electron *g*^⋆^ whose value includes corrections from the exchange energy^41^.

We find the effective *g*-factor to decrease from *g*^⋆^ = 6.5 when the system is uncoupled (with the resonator plane about 1.2 mm away; Fig. 2a,b) to *g*^⋆^ = 4.5 when the system is ultrastrongly coupled, with the resonator plane positioned 0.35 μm from the Hall bar surface (approximately 0.6 μm from the 2DEG), suggesting a strong reduction of the exchange energy corrections through the cavity-induced electron–electron potential.

**Fig. 3:**
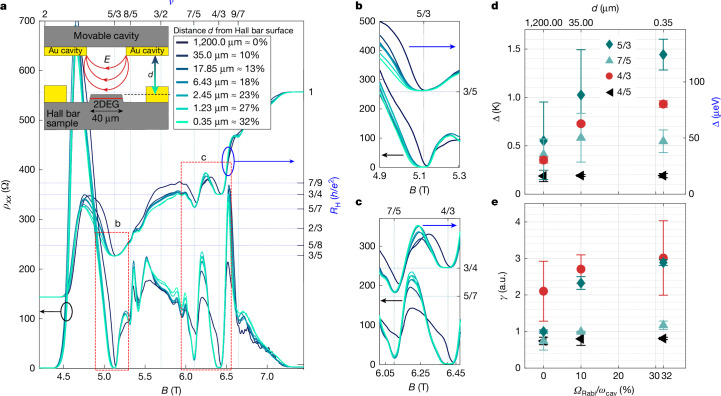
Cavity-enhanced fractional quantum Hall energy gaps. **a**, Longitudinal resistance (left vertical axis) and Hall resistance (right vertical axis) in the magnetic field region exhibiting the fractional quantum Hall states, for different distances *d* between the movable cavity and the Hall bar. Inset: side view schematic of the set-up. **b**,**c**, Zoom-in of the fractions 5/3 (**b**) and 7/5 and 4/3 (**c**). **d**, Activation energy gaps for fractional fillings 5/3, 7/5, 4/3 and 4/5 as a function of the normalized coupling *Ω*_Rabi_/*ω*_cav_ and corresponding distance between the split-ring resonator and the Hall bar. **e**, Decay rate *γ* of the power law dependence *ρ*_*xx*_ = *αT*^*γ*^ for the same fractions reported in **d**. The error bars in **d**,**e** indicate the 95% confidence interval of the weighted fit (Supplementary Information) of the Arrhenius law or, respectively, the power law. a.u., arbitrary units.

In contrast to the observed degradation of the quantization for the integer quantum Hall plateaux with increased light–matter coupling, the influence of the cavity vacuum fields on fractional quantum Hall states is profound and consists of an overall improvement of the quantization of the fractional states 5/3, 7/5 and 4/3, as depicted in Fig. 3a–c. This improvement was observed in a consistent manner in our temperature study and for the two resonators measured (Supplementary Information). At the lowest temperature, with progressively increased coupling, we observe a noticeable widening of the plateaux alongside a reduction in the resistance minima of *ρ*_*xx*_ (Fig. 3b,c).

**Extended Data Fig. 3:**
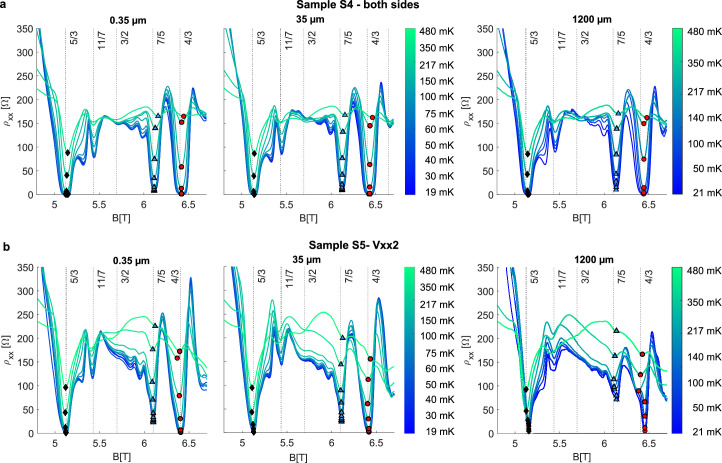
Transport measurements at different temperatures. **(a)** Temperature data taken on the Hall bar sample S4 coupled to the slot antenna and the extracted points ${\rho }_{\min }$ for the fractions 5/3, 7/5 and 4/3. At a distance *d* = 0.35 *μ*m and *d* = 35 *μ*m the longitudinal measurements were taken at 11 temperature points (measured at 0 T on the mK-plate of the cryostat). At the distance *d* = 1200 *μ*m (panels on the right) 7 temperature points were taken. **(b)** Same plots as in **(a)** for Hall bar sample S5 coupled to the CSRR.

**Extended Data Fig. 4:**
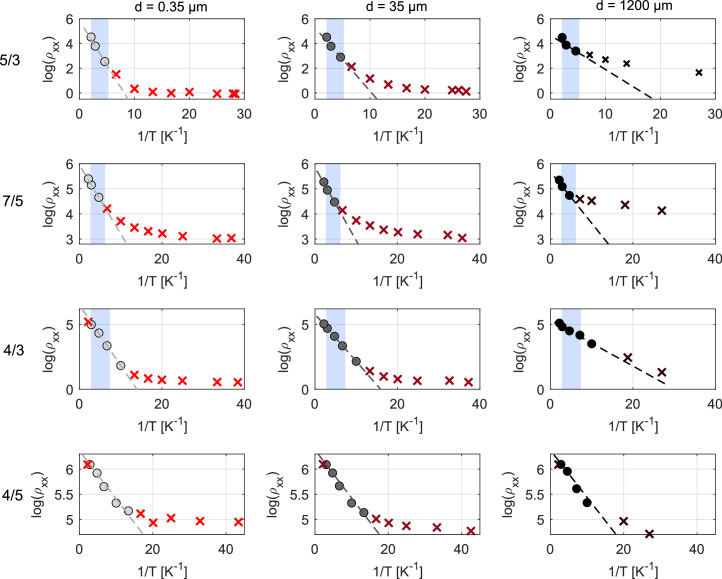
Sample S5, side Vxx2. Arrhenius plots for the fractional states 5/3, 7/5, 4/3 and 4/5 for the distances *d* = 0.35 *μ*m (left column), *d* = 35 *μ*m (middle column) and *d* = 1200 *μ*m (right column). Red crosses indicate points excluded from the weighted fit. The blue shaded area for the fractions 5/3, 7/5 and 4/3 indicates the temperature interval for which the activation temperature in the reference van der Pauw configuration was extracted (see Extended Data Fig. 1).

**Extended Data Fig. 5:**
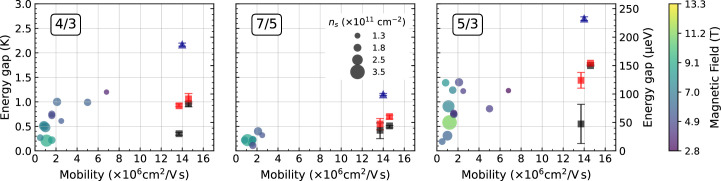
Energy gaps as a function of mobility for 4/3, 7/5, and 5/3 fractional fillings, comparing the results of the present work (black and red squares), with the measurement employing the van der Pauw method (blue triangles), and with data from refs. ^50–61^ (colored circles). The size of the circle markers indicates the sheet density of the sample on which the gap was measured (according to the legend in the middle panel), while their color indicates the magnetic field at which the corresponding fractional filling is reached (according to the colorbar on the right). The black squares refer to the case in which the resonator plane is 1200 *μ*m far from the Hall bar, while the red squares to the case in which the resonator plane is close to the 2DEG (0.35 *μ*m distance). The gaps measured via the van der Pauw method (blue triangles) are almost twice in magnitude with respect to the ones measured in the 40 *μ*m-wide Hall bar geometry.

We assess the energy gap by measuring the thermally activated longitudinal resistance (Extended Data Fig. 3), which exhibits a characteristic exponential dependence on the inverse temperature, as shown in the Arrhenius plots in Extended Data Fig. 4. As the distance is reduced from 1.2 mm to 0.35 μm, there is a noticeable increase in the energy gap for the fractional filling factors 5/3, 7/5 and 4/3 within the second Landau level (Fig. 3d). It is important to note that these fractions are part of the Jain principal sequence^29^ originating from the 1/3 Laughlin state. Although our highest magnetic field strength does not permit us to reach the filling factor *ν* = 2/3, we are able to achieve the fraction 4/5, which is associated with the 1/5 Laughlin state. The energy gap for the 4/5 fraction, observed in the lowest Landau level, does not increase significantly within the error bars. This suggests that the cavity-mediated effects may have contrasting impacts on the 1/3 and 1/5 families of fractional quantum Hall states. This behaviour is highlighted by extending the temperature analysis to a lower regime, in which the longitudinal resistivity *ρ*_*xx*_ follows a power law: *ρ*_*xx*_ = *αT*^*γ*^. The decay rate *γ* shows consistent behaviour for the fractions 5/3, 7/5 and 4/3, increasing with coupling, whereas the fraction 4/5 remains unaffected (Fig. 3e). Although the activation energy of 5/3 and 7/5 has large errors, the corresponding error in *γ* is small enough to clearly indicate variations beyond the error margin. These two methods of analysis on the same dataset confirm the cavity’s effect on the fractions. A detailed discussion of the complete temperature analysis is provided in the Supplementary Information. It is important to note that this analysis does not aim to provide a more precise value for the activation gap, but rather to highlight, through a self-referenced investigation, the intricate effects of the cavity on various states within the quantum Hall system. Comparison with reference measurements on the same heterostructure in van der Pauw geometry in Extended Data Fig. 5 shows that activation energies in those samples almost double compared to those measured in our relatively narrow Hall bars. Such dependence is expected as it is generally observed that the quality of the fractional states decreases with sample area^35^.

As fractional quantum Hall states arise solely from electron–electron interactions, these observations suggest that the cavity mediates an additional effective electron–electron interaction that competes with the Coulomb interaction. Such cavity-mediated interactions are also discernible at odd-integer filling factors, for which the activation energy gap is typically defined by spin splitting. In GaAs, this energy gap is predominantly influenced by the exchange splitting due to Coulomb interactions. The modifications in exchange splitting observed in our experiments also indicate the presence of a cavity-mediated potential.

To understand the cavity-mediated effects, we present here a simplified theoretical description for electrons subject to a perpendicular magnetic field and a single cavity mode (see the Supplementary Information for further details).

For the cavity mode, whose angular frequency is *ω*_cav_ and whose bosonic creation (annihilation) operator is ${\widehat{a}}^{\dagger }$ ($\widehat{a}$), we assume a linearly polarized vector potential with a constant spatial gradient, namely ${\widehat{{\bf{A}}}}^{{\rm{cav}}}(\widehat{{\bf{r}}})=({A}_{{\rm{vac}}}+{{\mathcal{G}}}_{{\rm{A}}}\,\widehat{y})(\widehat{a}+{\widehat{a}}^{\dagger }){{\bf{e}}}_{y}$. The gradient ${{\mathcal{G}}}_{{\rm{A}}}$ is related to the gradient of the vacuum electric field through ${{\mathcal{G}}}_{{\rm{E}}}={\omega }_{{\rm{cav}}}{{\mathcal{G}}}_{{\rm{A}}}$. The quantum light–matter interaction consists of a paramagnetic contribution (linear in the photon operators) and a diamagnetic term (quadratic in the bosonic operators). The part of the Hamiltonian that depends on the photonic operators alone can be diagonalized through a Bogoliubov transformation in terms of the renormalized boson operator $\widehat{\alpha }$ and cavity mode frequency ${\widetilde{\omega }}_{{\rm{cav}}}$ (ref. ^17^).

**Fig. 4:**
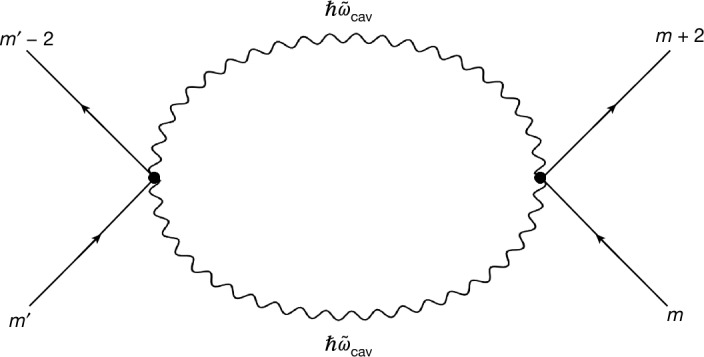
Effective interaction vertex. Diagram representing the exchange of two angular momenta between distant electrons through the exchange of two cavity photons.

If the cavity photon field is not resonant to the electronic transitions, we can adiabatically eliminate it and determine an effective electron–electron interaction within a given Landau band (Supplementary Information and D.B. et al., manuscript in preparation):

1 $${\widehat{{\mathcal{H}}}}_{{\rm{e}}-{\rm{e}}}^{({\rm{cav}})}\simeq -\frac{{{\mathcal{D}}}^{2}}{4\hbar {\widetilde{\omega }}_{{\rm{cav}}}}\sum _{{m}_{1}{m}_{2}}{\eta }_{{m}_{1}{m}_{2}}{\widehat{c}}_{{m}_{2}+2}^{\dagger }{\widehat{c}}_{{m}_{1}-2}^{\dagger }{\widehat{c}}_{{m}_{1}}{\widehat{c}}_{{m}_{2}},$$

with ${\eta }_{{m}_{1}{m}_{2}}=\sqrt{{m}_{1}}\sqrt{{m}_{1}-1}\sqrt{{m}_{2}+1}\sqrt{{m}_{2}+2}$ and ${\mathcal{D}}=\left(\frac{{\omega }_{{\rm{cav}}}}{{\widetilde{\omega }}_{{\rm{cav}}}}\right){({{\mathcal{G}}}_{{\rm{A}}}{\ell })}^{2}\left(\frac{{e}^{2}}{2{m}_{\star }}\right)$, in which ${\ell }=\sqrt{\hbar /eB}$ is the magnetic length, −*e* is the electron’s charge, and *m*_⋆_ is its effective mass. The fermionic operator ${\widehat{c}}_{m}^{\dagger }$ creates an electron in the state with angular momentum *m* (symmetric gauge) in the considered Landau level (Fig. 4). For simplicity, we are omitting the spin index. Note that the spatial gradient is essential to create an intraband interaction.

We have been able to calculate analytically the effect of ${\widehat{{\mathcal{H}}}}_{{\rm{e}}-{\rm{e}}}^{({\rm{cav}})}$ on the exchange spin splitting at odd-integer filling factors and also on the modification of fractional quantum Hall gaps in the so-called single-mode approximation^42^. The cavity contribution to the exchange splitting is $\langle {\rm{FP}}| {\widehat{{\mathcal{H}}}}_{{\rm{e}}-{\rm{e}}}^{({\rm{cav}})}| {\rm{FP}}\rangle$, in which $| {\rm{FP}}\rangle ={\widehat{c}}_{0}^{\dagger }\ldots {\widehat{c}}_{{N}_{\text{deg}}-1}^{\dagger }| {\rm{vac}}\rangle$ is the fully packed state corresponding to an odd-integer filling factor. When the Landau degeneracy *N*_deg_ ≫ 1, we have $\Delta {E}^{{\rm{exc}}}\simeq \langle {\rm{FP}}| {\widehat{{\mathcal{H}}}}_{{\rm{e}}-{\rm{e}}}^{({\rm{cav}})}| {\rm{FP}}\rangle \simeq \frac{{{\mathcal{D}}}^{2}}{12\hbar {\widetilde{\omega }}_{{\rm{cav}}}}{N}_{\text{deg}}^{3}$, in which ${N}_{\text{deg}}=\frac{{L}_{x}{L}_{y}}{2{\rm{\pi }}{{\ell }}^{2}}$ with *L*_*x*_ and *L*_*y*_ being the spatial dimensions of the 2D sample. As the effective *g*-factor is dominated by Coulomb interactions, and the cavity-mediated potential is attractive, the cavity correction to *g*^⋆^ (the absolute value of the *g*-factor) is hence negative:

2 $$\Delta {g}^{\star }=-\frac{1}{{N}_{\text{deg}}}\frac{\Delta {E}^{{\rm{exc}}}}{{\mu }_{{\rm{B}}}B}\simeq -\frac{1}{48}\frac{{e}^{4}}{\hbar {\mu }_{{\rm{B}}}B{m}_{\star }^{2}}\frac{1}{{\widetilde{\omega }}_{{\rm{cav}}}^{5}}{{\mathcal{G}}}_{{\rm{E}}}^{4}{{\ell }}^{4}{N}_{\text{deg}}^{2},$$

in which *μ*_B_ is the electron Bohr magneton. Let us consider parameters corresponding to our experimental configuration, namely ${\widetilde{\omega }}_{{\rm{cav}}}=7.5\times 1{0}^{11}\,{\rm{rad}}\,{{\rm{s}}}^{-1}$, a density of electrons *n* = 2 × 10^11^ cm^−2^, *L*_*x*_ = *L*_*y*_ = 100 × 10^−6^ m and *B* = 0.5 T, and with the spatial gradient of the vacuum electric field being ${{\mathcal{G}}}_{{\rm{E}}}=4\times 1{0}^{8}\,{{\rm{V}}{\rm{m}}}^{-2}$. With these values, we get Δ*g*^⋆^ ≃ −2, which is close to what is observed in the experiment.

To describe fractional quantum Hall states, for any two-body interaction that conserves angular momentum, the Haldane pseudo-potential components are key quantities^20^. We have found the following long-range Haldane pseudo-potentials for the cavity-mediated interaction: ${v}_{m}^{{\rm{(cav)}}}=\left(-\frac{{{\mathcal{D}}}^{2}}{8\hbar {\widetilde{\omega }}_{{\rm{cav}}}}\right)({m}^{2}-m)$. We remark that if only Coulomb interaction (${v}_{m}^{({\rm{C}})}\propto 1/\sqrt{m}$) were present, its short-range component ${v}_{1}^{({\rm{C}})}$ would be responsible for the FQH gap opening, whereas the long-range ones ${v}_{m > 1}^{({\rm{C}})}$ would reduce the magnitude of the gap opened by ${v}_{1}^{({\rm{C}})}$. Our cavity-mediated interaction, which is added on top of Coulomb interaction, leaves the short-range components unaffected, and decreases the long-range components; thus, an overall increase of the gap would not be unexpected (see Supplementary Information for exact diagonalization results).

An interaction potential corresponding to the cavity-mediated potential Haldane components $${v}_{m}^{({\rm{cav}})}$$ reads: $${V}^{({\rm{cav}})}(r)\,=$$$$\left(-\frac{{{\mathcal{D}}}^{2}}{8\hbar {\widetilde{\omega }}_{{\rm{cav}}}}\right)\left[\frac{1}{16}{\left(\frac{r}{{\ell }}\right)}^{4}-{\left(\frac{r}{{\ell }}\right)}^{2}+2\right]$$. Note that the divergence at large distances *r* is due to the approximation of considering a cavity mode with a constant spatial gradient $${{\mathcal{G}}}_{{\rm{E}}}$$. A cutoff at large distances is not only expected by the finite-size nature of the Hall bar but also due to the fact that the spatial region with large gradients is a fraction of the sample (Fig. 1). To evaluate the many-body gap of fractional quantum Hall states, a powerful technique is the Girvin–MacDonald–Platzman magneto-roton theory^42^, which assumes that the collective excitation gap can be determined by the sole knowledge of the (Laughlin) ground state. The Girvin–MacDonald–Platzman model is well known to faithfully capture the minimum of the composite-fermion exciton magneto-roton mode on top of the Laughlin states, giving an approximate lower bound for the large-*k* energy of this mode, which corresponds to the transport gap^43^.

Considering the potential *V*^(cav)^(*r*) provides a divergent result. We have regularized that by introducing an infrared cutoff due to the finite size of the sample.

The variation of the fractional quantum Hall gap due to the presence of the cavity reads

3 $${\Delta }^{({\rm{cav}}+{\rm{C}})}-{\Delta }^{({\rm{C}})}\simeq 0.04{\left(\frac{L}{{\ell }}\right)}^{4}\frac{{{\mathcal{D}}}^{2}}{8\hbar {\widetilde{\omega }}_{{\rm{cav}}}}\frac{{({\ell }{k}_{\min })}^{2}{e}^{-\frac{1}{2}{({\ell }{k}_{\min })}^{2}}}{\overline{s}({k}_{\min })}\frac{\nu }{8{{\rm{\pi }}}^{2}},$$

in which *L* is the length associated with the infrared cutoff (see Supplementary Information for more details). Note that, as in the case of the cavity-modified *g*-factor, the variation of the fractional quantum Hall gap scales with ${{\mathcal{D}}}^{2}{L}^{4}\propto {{\mathcal{G}}}_{{\rm{E}}}^{4}{N}_{\text{deg}}^{2}$, showing again the key role of the spatial gradient and the collective electron contribution due to the long-range nature of the perturbation. By taking *L* = 100 μm (roughly the system size) and spatial gradients of the same order as the ones used for the calculation of the exchange splitting, setting ${\ell }{k}_{\min }=1.3$ and $\overline{s}({k}_{\min })\approx 1$ (the values for the 1/3 Laughlin state^42^), we get $\frac{{\Delta }^{({\rm{cav+C}})}-{\Delta }^{({\rm{C}})}}{{\Delta }^{({\rm{C}})}}\approx 0.5$, which is comparable to what we observed in our experiments. We remark that the states 4/3 and 5/3, in the absence of spin mixing and Landau level mixing, are the 1/3 state and its conjugate hole, 2/3, in the top Zeeman-split lowest Landau level. Although the already complex theory is based on many simplifications (single-mode cavity, constant spatial gradient of the vacuum electric field and magneto-roton theory for the excitation gap), the magnitude of the predictions is consistent with the experimental observations.

This joint experimental and theoretical study has demonstrated the capacity of electromagnetic vacuum fields with pronounced spatial gradients to control complex, strongly correlated electronic systems, specifically fractional quantum Hall phases. Notably, we observed a significant enhancement of fractional quantum Hall gaps within a key group of fractions. We have revealed that the exchange of virtual cavity photons can induce an effective attractive long-range electron–electron interaction, which competes with the Coulomb interaction. Additionally, we found that this cavity-mediated attractive interaction substantially reduces the exchange spin splitting, which is responsible for the activation energy gap at odd-integer filling factors.

These experimental results were achieved using a ‘hovering’ resonator technique. This method finely tunes the electromagnetic vacuum fields by adjusting the distance between the cavity and the Hall bar, offering precise control over the coupling strength. Moreover, this approach has broader implications and can be generalized to any 2D material and small planar quantum device. Specifically, leveraging cavity vacuum fields in this manner could manipulate strongly correlated phases in moiré materials, such as by stabilizing fractional Chern insulators^44–46^ or altering superconductivity^47^ properties, and may ultimately be implemented within quantum information processing purposes.

*Note added in proof:* While completing this manuscript, we became aware of a theoretical preprint focusing on the resonant hybridization of the magneto-roton with the cavity photon field^48^.

## Methods

### Sample design

**Extended Data Fig. 6:**
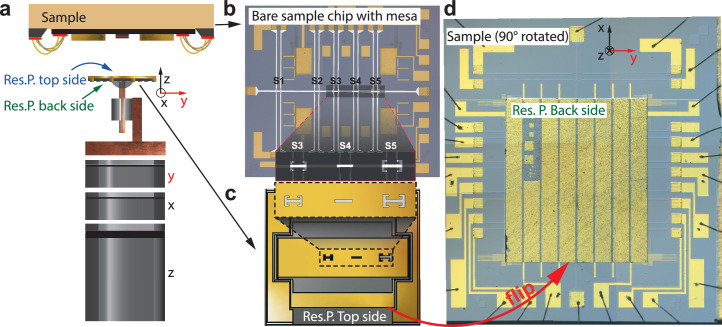
Extended setup description. **(a)** Sketch of the sample chip mounted top down in the cryostat with the movable resonator plane (Res.P.), top side facing up, mounted from below with a copper rod attaching it to three nanopositioners moving in x, y and z direction. **(b)** Optical microscope picture of the chip with the etched mesa structure (highlighted in white) and all of the Hall bar samples S1 to S5. Zoom in red frame: depiction of how the CSRR on the resonator plane and the Hall bar are designed to overlay. **(c)** Drawing of the resonator plane design with zoom-in to the CSRR. **(d)** Optical microscope picture of the pre-aligned sample (90° rotated clockwise with respect to **(b,c)**): in this view the resonator plane has already been flipped. The thick gold lines on the back side of the resonator plane serve as alignment markers that line up with gold markers on the sample chip.

On a 6.3 × 6 mm^2^ chip of the 2DEG material, we define a 5,376-μm-long, 40-μm-wide and 350-nm-thick mesa with probes leading to five different positions, S1, S2, S3, S4 and S5 (Extended Data Fig. 6b). Probes leading to the samples have a length of 2 mm and a width of 30 μm and connect to the Hall bar with a tapered width of 10 μm. This mask layout allows us to probe multiple Hall bar samples on the same mesa at the same time. The sample shown in Extended Data Fig. 6b has four pillar-shaped landing pads (the big gold rectangles between S1 and S2, and after S5) that probe the electrical connection between the resonator plane and the Hall bar sample and serve as a safeguard against damaging the Hall bar with the movable hovering resonator plane. Indeed, when the gold resonator plane is in contact with the pillars, it electrically shorts the connection between them, thus allowing the touching position to be confirmed from outside the cryostat.

The resonator plane consists of a 3,620 × 3,200 μm^2^ piece of insulating GaAs, lapped down to a thickness of 150 μm. On the top side of the resonator plane (Extended Data Fig. 6c), we lithographically define the three CSRRs and two electrically disconnected gold planes at the corners, which are electrically connected via evaporated gold on the substrate edges to the back side of the resonator plane chip. As seen in Extended Data Fig. 6d, which shows a picture of the top view of the pre-aligned Hall bar sample with the resonator plane (here back side view), alignment markers on both the back side of the resonator plane (lifted-off lines) and the Hall bar sample guarantee the spatial overlap of the samples S3, S4 and S5 with the resonators (Extended Data Fig. 6b, bottom). The uncoupled fundamental mode of the CSRR on S3 is engineered to resonate at a frequency of 150 GHz, the one on S4 at 200 GHz, and the one on S5 at 130 GHz. The resonator gaps of the CSRRs overlapping with S3 and S4 have a width of 40 μm, making the alignment precision a crucial factor, whereas the gap of the CSRR overlapping with S5 spans a width of 50 μm.

### Alignment of the Hall bar with the cavity

**Extended Data Fig. 7:**
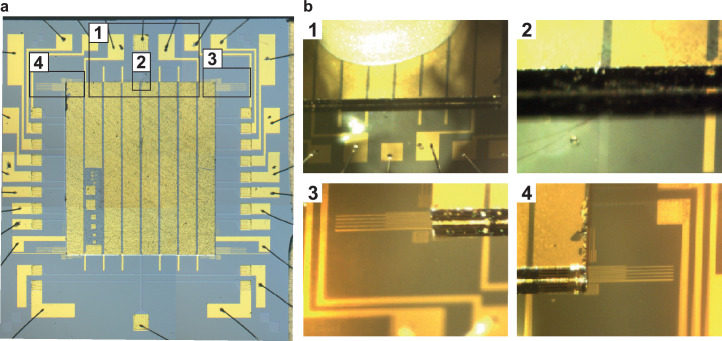
Close-up photographs detailing the alignment. **(a)** Optical microscope picture of pre-aligned structure: sample chip below the upside-down resonator plane. **(b)** Digital camera pictures of the alignment after mounting on the cryostat cold finger. In B1, one can see the glue with which the copper rod gets attached to the back side of the resonator plane. B2 shows the precise alignment of the 40 *μ*m-wide Hall bar to the marker to the back of the resonator plane. B3 and B4 show close-up pictures on the edges of the resonator plane where, with alignment markers forming a grid, last precise alignment adjustments can be made. The angular alignment is also ensured via this procedure.

A critical factor of this experiment is the correct alignment of the resonator plane with the Hall bar samples defined on the mesa structure on the sample chip. Before transferring the chip into our Bluefors dilution refrigerator, we pre-align the sample and the resonator plane in a clean-room environment. First, we place a droplet of water on the surface of the Hall bar sample, then place the resonator plane (with the CSRRs facing the Hall bar) on top of the droplet, and then align the resonator plane using a micromanipulator. Finally, we gently press the resonator plane on top of the sample chip, so that the van der Waals force of the water droplet holds the resonator plane in place. Then, the pre-aligned geometry, as shown in Extended Data Fig. 7a, can be transferred onto the cold finger in the dilution fridge. At room temperature, the resonator plane is glued with conductive epoxy to a copper rod, which is mounted on a stack of piezoelectric attocube nanopositioners, which can move in the *x*, *y* and *z* directions. After the glue is dry, we perform the last alignment procedures before cooling the system down. In Extended Data Fig. 7b, we show close-up pictures of the alignment of the markers of the resonator plane and sample chip, already mounted on the dilution fridge cold finger at room temperature. Any residual water will be removed by pumping during the cool-down process.

### Conducting the experiment

This study utilizes state-of-the-art techniques for analysing the quantum Hall effect in 2D electron systems^35^. Using a Bluefors dilution refrigerator, we can cool to temperatures as low as 19 mK. Voltage measurements are carried out using Zurich Instruments MFLI digital lock-in amplifiers. To inject current symmetrically into the mesa structure, an a.c.-modulated voltage of 2 V (r.m.s.) at a demodulation frequency of 2.333 Hz is applied across two 100-MΩ resistors in series with the mesa, generating a current of 10 nA (r.m.s.). Differential a.c. low-noise pre-amplifiers are used to amplify the signal by a factor of 1,000 before it reaches the lock-in voltage input. A fourth-order low-pass filter with a time constant of 1.0 s is used to demodulate the input signal to the lock-in amplifier. Additionally, low-pass 100-kHz filters are installed before the sample contacts to reduce electrical spikes or potential heating effects from the measurement set-up.

As shown in Extended Data Fig. 6a, the three piezoelectric actuators can be moved to tune the coupling between the resonator plane and each Hall bar sample. In this study, we conducted thorough measurements only on the samples S4 and S5. Immediately after cooling down, the resonator plane is brought into contact with the pillar-shaped landing pads on the chip. When a short between all four pillars is measured, we can guarantee a parallel alignment of the resonator plane to the surface of the Hall bar sample, an initial distance of ≈450 nm from the 2DEG, and the equilibrium of the electrochemical potentials of the resonator plane and of the Hall bar sample. To convert the number of retraction steps of the piezoelectric actuators from this initial position, we measure the distance between 0 and 200 retracted steps of the piezoelectric actuators at 4 K as the position reading of the piezoelectric actuators is limited at millikelvin temperatures. We point out that indeed thermal expansion might shift the precision of the estimated position.

The set-up permits us to measure the longitudinal and transverse resistance of samples S4 and S5 at the same time. Each trace of longitudinal and transverse voltage was measured for a specific distance between the surface of the resonator plane and the surface of the Hall bar as a function of magnetic flux density, which is tuned by means of a superconductive solenoid magnet capable of reaching 12 T. We point out that for the measurement performed at a distance 0.35 μm, the Hall bar sample and the resonator plane are not touching and are electrically disconnected from each other.

## Online content

Any methods, additional references, Nature Portfolio reporting summaries, source data, extended data, supplementary information, acknowledgements, peer review information; details of author contributions and competing interests; and statements of data and code availability are available at 10.1038/s41586-025-08894-3.

## Supplementary information

Supplementary Information Supplementary Sections 1–7, including Figs. 1–19 and References.

## Data Availability

Data supporting this article’s findings are available in the ETH Research Collection^49^.
