# Supplementary material for: Tunable vacuum-field control of fractional and integer quantum Hall phases
Source: Nature. 2025 May 14;641(8064):884–9. doi: 10.1038/s41586-025-08894-3 (PMC12095043; doi:10.1038/s41586-025-08894-3)
Supplement: Supplementary file 1 — Supplementary Sections 1–7, including Figs. 1–19 and References. [file 41586_2025_8894_MOESM1_ESM.pdf]

nature portfolio

https://doi.org/10.1038/s41586-025-08894-3

Supplementary information

Tunable vacuum-field control of fractional and integer quantum Hall phases

In the format provided by the authors and unedited

Supplementary Material for:
Tunable vacuum-field control of fractional and integer quantum Hall phases

Josefine Enkner*,[1,2] Lorenzo Graziotto,[1,2] Dalin Boriçi,[3] Felice Appugliese,[1,2] Christian Reichl,[4]
Giacomo Scalari,[1,2] Nicolas Regnault,[5,6,7] Werner Wegscheider,[4] Cristiano Ciuti,[3] and Jérôme Faist*[1,2]

[1] *Institute for Quantum Electronics, ETH Zurich, CH-8093 Zurich, Switzerland*
[2] *Quantum Center, ETH Zürich, 8093 Zürich, Switzerland*
[3] *Université Paris Cité, CNRS, Matériaux et Phénomènes Quantiques, 75013, Paris, France*
[4] *Laboratory for Solid State Physics, ETH Zürich, 8093 Zürich, Switzerland*
[5] *Center for Computational Quantum Physics, Flatiron Institute, 162 5th Avenue, New York, NY 10010, USA*
[6] *Laboratoire de Physique de l'Ecole normale supérieure, ENS, Université PSL, CNRS, Sorbonne Université*
[7] *Department of Physics, Princeton University, Princeton, NJ 08544, USA*

This Supplementary Material concerns both the experiments and theory described in the main manuscript. In particular, it provides additional details concerning the setup, sample, data analysis and measurement techniques. On the theoretical side, it details the Hamiltonian of the system and the derivation of the effective interaction, contains sections showing the negligible role of electrostatic effects, some exact diagonalization results for a small number of electrons, and details about the magneto-roton theory of the fractional quantum Hall gaps that can be applied for extensive systems.

CONTENTS

I. Material and Methods 2
A. Material details on the Hall bar heterostructure and the resonator plane 2
B. Fabrication of the Hall bar sample and the resonator plane 2

II. Finite element simulations 3

III. Additional Data & Analysis 5
A. Integer quantum Hall plateaus analysis for the CSRR resonator (S5) 5
B. Temperature-dependent resistivity and activation gaps 6
C. Measurement of the g-factor on resonator S4 6
D. Fractional states 8
1. Sample S4 - combining the data of both sides Vxx1 and Vxx2 8
2. S4 and S5: evaluation of the cavity-driven improvement of the quantization 8
3. Magnetotransport curves for different distances as a function of temperature for both Hall bars S4 and S5 9
4. Determination of the gap for the fractional states 9
5. Results Temperature Analysis for S4 and S5 11
6. Measurement of the fractional gaps with van der Pauw's method and comparison with literature data 13

IV. Hamiltonian Formalism 16
A. Hamiltonian of the 2DEG in a single mode cavity 16
B. Effective Interaction 16

V. Negligible role of electrostatic screening on Coulomb potential from distant hovering resonator 17
A. Experimental aspects 17
B. Theoretical aspects 17

VI. Exact diagonalization result with an effective cavity-mediated potential 18

VII. Magneto-roton theoretical results for the fractional quantum Hall gaps 19

References 21

I. MATERIAL AND METHODS

A. Material details on the Hall bar heterostructure and the resonator plane

The GaAs-based heterostructure D151202B employed for the Hall bar was grown via molecular beam epitaxy (MBE) by Dr. Christian Reichl in the Solid State Laboratory at ETH Zürich. The D151202B hosts a double-side doped, 30 nm wide GaAs/AlGaAs quantum well with a 100 nm spacer and is buried 233 nm under the surface. Due to the double-side doped design of the quantum well, the sample is immune to gate-induced density and mobility fluctuations at low electric fields. The material exhibits a high electron mobility 1.69×10^7 cm^{2}V^{-1}s^{-1} and sheet density 2.06×10^{11} cm^{-2}, measured at 1.3 K without illumination. The resonator plane consists of a square 3620×3200 µm^2 piece of insulating GaAs, lapped down to a thickness of 150 µm, on top of which the complementary split-ring resonator (CSRR) is fabricated.

B. Fabrication of the Hall bar sample and the resonator plane

Hall bar sample: Starting from the heterostructure, grown by MBE, the fabrication of the sample is carried out by optical lithography. The steps are the following:

- Definition of the Hall bar using positive resist (AZ1505) and photolithography;
- Etching of the mesa using a very diluted Piranha acid (H_2SO_4:H_2O_2(30%):H_2O 1:8:60);
- Contacts are defined photolithographically using a negative image-reversal resist (AZ5214E);
- Ge/Au/Ge/Au/Ni/Au (41/84/41/84/63/40 nm) eutectic mixture evaporation, lift off and annealing (500°C for 300 s);
- Extra gold pads are defined again by optical lithography with image-reversal resist;
- Deposition of Ti/Au (11/200 nm) and lift off.

Resonator Plane: Starting from a 1×1 cm^2 piece of insulating GaAs, we define 9 resonator plane structures that, during the process, will get cleaved and processed separately:

- Definition of the cleaving guides using a positive resist (AZ1505) and photo-lithography;
- Etching of the cleaving guides using a very diluted Piranha acid (H_2SO_4:H_2O_2(30%):H_2O 1:8:60);
- Resonator plane is defined photolithographically using a negative image-reversal resist (AZ5214E);
- Deposition of Ti/Au (11/200 nm) and lift off;
- Spinning protective layer of resist;
- Lapping the sample down to a thickness of 150 µm;
- Definition of the alignment markers using a negative resist (AZ5214E) and photo-lithography on the back of the chip;
- Deposition of Ti/Au (11/200 nm) and lift off;
- Spinning protective layer of resist on both sides;
- Cleaving the GaAs sample into 9 separate resonator planes along the cleaving guides;
- Deposition of Ti/Au (11/200 nm) on the side walls and lift off.

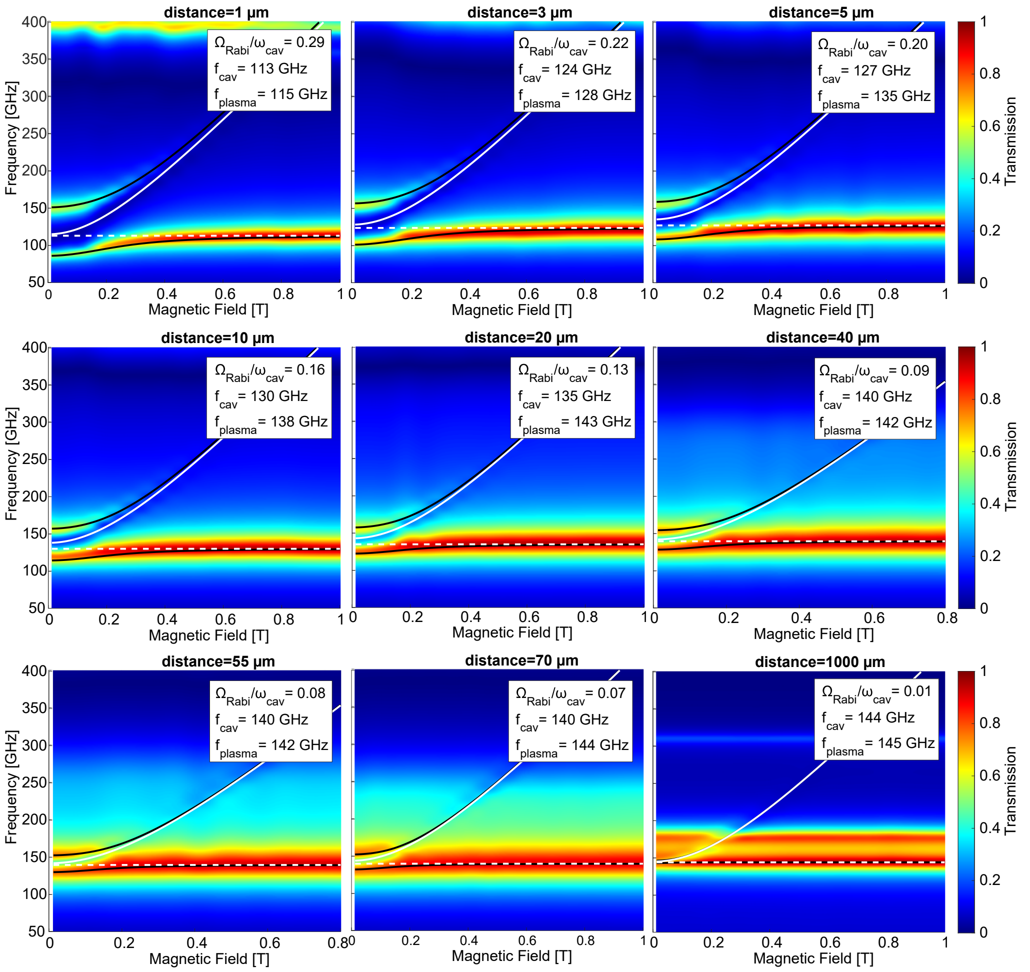

FIG. S1. Simulation of the polaritonic energy dispersions for different distances between resonator plane and the 2DEG. In the legends the parameters of the fit to the Hopfield model are reported.

II. FINITE ELEMENT SIMULATIONS

In the following we discuss supporting material on the finite element simulations performed in order to estimate the normalized coupling $\Omega_{\mathrm{Rabi}}/\omega_{\mathrm{cav}}$[1] of the system and the field profile of the vacuum electric field E_{vac} inside the Hall bar as a function of distance between the 2DEG and the CSRR. All the simulations of Figure S1 and Figure S2 are performed using the CST Microwave Studio software.

The resonator plane is modeled using the standard lossy metal gold from the material library on top of a GaAs substrate. Similarly, the substrate of the Hall bar sample is a block of GaAs. The 2DEG stripe is modeled using a gyrotropic material with bias (i.e., the magnetic field) in the direction perpendicular to the surface. An effective layer thickness was used in order to reduce computational cost. The distance between the 2DEG and the CSRR is tuned parametrically. In Figure S1, we show the polaritonic dispersion obtained from the s-parameter parallel to the excitation field (perpendicular to the resonator gap) from the simulations for different values of the distance between the 2DEG and the resonator plane. Fitting the Hopfield model[2] on top of the dispersion, we estimate the normalized coupling that is also used at a later point to calculate the vacuum electric field penetrating the Hall bar. This method tends to overestimate the coupling strength but offers a reliable verification of the expected trend for the resonators, which have been measured optically using our THz time-domain spectroscopy set-up described in Refs.[3–5].

The coupling can be estimated from the dispersion obtained from the simulation when sweeping the magnetic field and obtaining the magnetoplasma-polariton dispersion. This can be fitted with the Hopfield dispersion model[2], which will give us the normalized coupling strength g. From the coupling strength g we can estimate the coupled vacuum

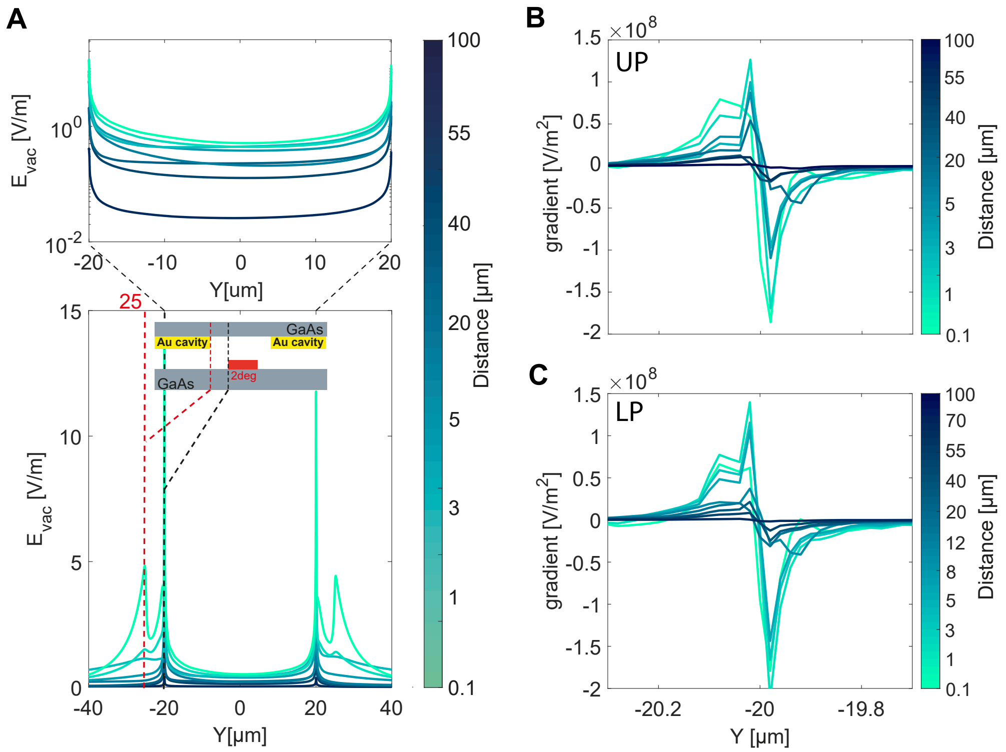

FIG. S2. **Field profile and field gradient at 0.3 T (A)** Field profile of the vacuum electric field across the 2DEG. Top: Zoom to the area within the Hall bar. **(B)** Field gradient $\mathcal{G}_E$ of the upper polariton (UP). **(C)** $\mathcal{G}_E$ of the lower polariton (LP).

field with

$$\mathcal{E}_{\text{vac}} = \frac{\hbar\Omega_{\text{Rabi}}}{d\sqrt{N_e}}$$

where $\Omega_{\text{Rabi}} = g\omega_{\text{cav}}$ is the Rabi frequency of the system, d is the dipole moment, and N_e is the number of coupled dipoles. The vacuum electric field $\mathcal{E}_{\text{vac}}$ estimated here is a constant, which is not true for the real vacuum field distribution within the gap of the resonator. For example, due to the interaction with the electrons in the 2DEG, the electric field profile has a very high amplitude at the interface between the vacuum and Hall bar edge and then decays into the bulk of the Hall bar (see the shape of the vacuum electric field in S2). However, we can take the estimated value of $\mathcal{E}_{\text{vac}}$ to normalize the electric field $E_{\text{sim}} = \sqrt{E_x^2 + E_y^2 + E_z^2}$ (total electric field strength) obtained in the simulation in the following fashion

$$E_{\text{vac}} = E_{\text{sim}} \cdot \sqrt{\left(\frac{\mathcal{E}_{\text{vac}}^2 \cdot W}{\int_{a_1}^{a_2} E^2}\right)} = E_{\text{sim}} \cdot \frac{\mathcal{E}_{\text{vac/rms}}}{E_{\text{sim/rms}}} \tag{S1}$$

where W indicates the Hall bar's width and $a_{1,2}$ are the boundaries of the Hall bar. Here, we effectively take the ratio of the root-mean-square values of the constant field $\mathcal{E}_{\text{vac}}$ over the electric field obtained in the simulation E_{sim} integrated over the Hall bar with width W (with boundaries $a_{1,2}$). This renormalization is necessary since the simulation is performed by exciting the structure (2DEG and resonator) with an electromagnetic wave. This does not correspond to our experimental conditions, where we measure the system without illumination at mK temperatures. Still, the estimation of the normalized coupling holds since, in the ultrastrong coupling regime, the coupling is independent of the number of real photons injected and only depends on the vacuum electric field $\mathcal{E}_{\text{vac}}$. Therefore, the polariton dispersion is still accurately reproduced in the simulation.

In Figure S2 we show the field profile simulation of the upper polariton (UP) within the Hall bar. Simulations were performed on the coupled system at a field corresponding to $B = 0.3\,\text{T}$. Similarly to the field profile shown in the main text, we can again identify four peaks symmetrically centered around zero. Two coincide with the edges of the cavity gap defined by the CSRR and two mark the boundaries of the Hall bar. We point out that the amplitude of

the peaks at ±25 µm is lower than for the lower polariton (LP), which can be explained due to the fact that the lower polariton frequency is closer to the cavity frequency. We note that the constant field component inside the Hall bar (Figure S2A top) is of the order of $E_{\mathrm{vac}} = 0.8$ V when the cavity is close and then decreases with increasing distance. We point out, however, that the gradient $\mathcal{G}_E$ for both lower and upper polaritons is of the order of 10^8 V/m^2, and we have employed this value to obtain numerical estimates from the theoretical model.

III. ADDITIONAL DATA & ANALYSIS

The investigation of the influence of light-matter coupling onto the transport in the integer and fractional quantum Hall effect relied on systematic magnetotransport experiments performed as a function of both temperature and resonator distance from the sample. To achieve highly accurate resistance measurements while maintaining a low temperature, each magnetic field sweep had a duration of about ten hours. As described above, the sample comprised a number of Hall bars (named S1–S5), two of which had a resonator, labelled S5 for the one covered by a CSRR and S4 for the one with the slot antenna. The main text reports the data measured on S5 (CSRR) where the effects were seen to be the most striking. In this section, we discuss and interpret additional data measured both on the Hall bar with a CSRR resonator (S5), displayed in the main text, and on the Hall bar coupled to a 40 µm-wide slot antenna resonator (S4), which possesses a resonance frequency of $\omega_{\mathrm{slot}} = 2\pi \times 200$ GHz. The maximal coupling for this resonator is estimated to be lower than the one of the CSRR (discussed in the main text) due to the higher resonance frequency.

A. Integer quantum Hall plateaus analysis for the CSRR resonator (S5)

To assess the influence of the cavity on the quality of quantization also for even denominator quantum Hall states where the longitudinal resistance remains quantized, we conducted the following analysis, as illustrated in Figure S3. Each plateau was modeled using a third-order polynomial fit $f(x) = \mathrm{p1}\cdot x^3 + \mathrm{p2}\cdot x^2 + \mathrm{p3}\cdot x + C$. The differences in fits for various distances between the resonator plane and the Hall bar sample are observable in the inset of Figure S3.

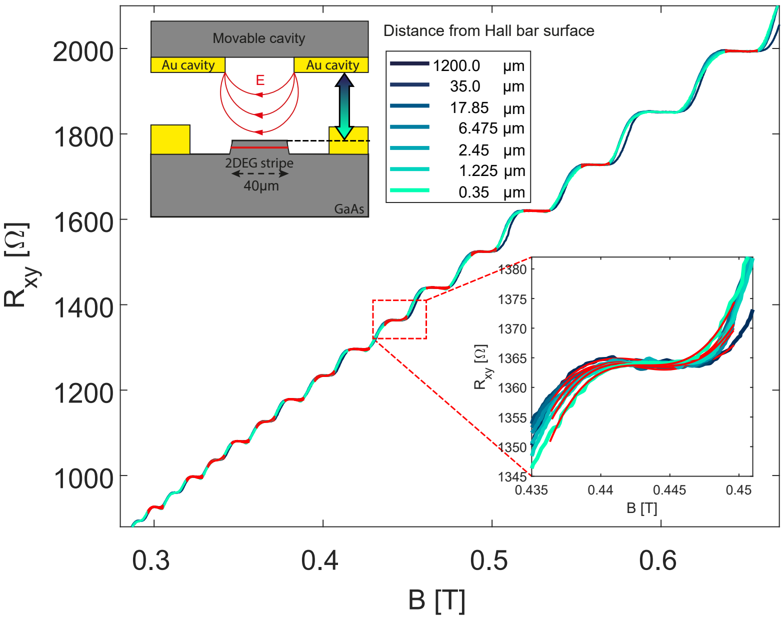

FIG. S3. Transverse resistance measured on Hall bar S5 for multiple distances between CSRR and Hall bar sample, and polynomial fit (red curves) around the plateau regions. Inset: zoom in to the plateau of filling factor 22 and fit (red curves) on top.

Subsequently, we plotted each polynomial component against the cyclotron energy, or equivalently as a function of the filling factor. This approach not only revealed a function that appears to decay exponentially as a function of cyclotron energy, but also showed a correlation between the decay rate and the distance between resonator plane and

Hall bar sample at which the measurements were taken (see Figure S4A,B). The observed exponential decay in this study mirrors the results found in Ref.[5], which reported a similar exponential decrease in cavity-induced resistivity. In that work, the characteristic energy of the exponential decay was identified as $E_{\mathrm{char}} = 0.38$ meV. Here, we look at the linear component of the polynomial fit p3 as a function of cyclotron energy. Our analysis identifies a characteristic energy (E_{char}) of 0.4 meV when the resonator plane is proximate, and 0.1 meV when it is distant (see Figure S4C). This analysis not only allows us to quantify the impact of the cavity on both odd and even plateaus but also allows us to corroborate the previous findings of Ref.[5].

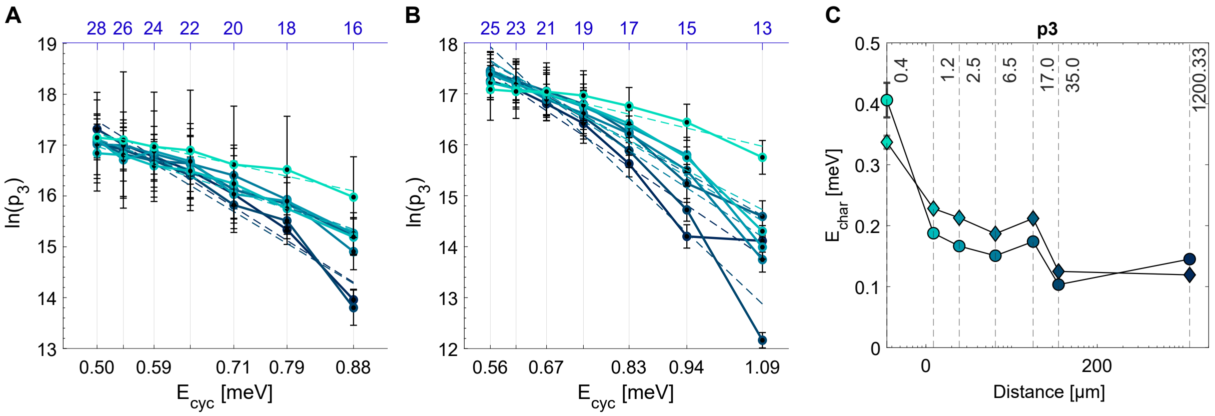

FIG. S4. **Exponential decay of the linear fitting parameter p3 (A)** Exponential decay of the linear fitting parameter p3 for even filling factors 28 to 16 as a function of cyclotron energy E_{cyc}. Each colored line corresponds to the linear fitting value p3 obtained for a specific distance, following the color scheme in the legend of Fig. S3: light color is close (0.35 µm) while dark is far away (1200 µm). **(B)** Exponential decay of the linear fitting parameter p3 for odd filling factors 25 to 13 as a function of E_{cyc}. **(C)** Characteristic energy E_{char}, associated with the exponential decay of p3 as a function of E_{cyc}, as a function of distance. Diamonds (circles) refer to even (odd) filling factors.

B. Temperature-dependent resistivity and activation gaps

Arrhenius law: By measuring the longitudinal resistance ρ_{xx} at various temperatures, as shown for the integer states in Figure S6A and the fractional states in Figure S9, one can estimate the energy gaps of these states. The temperature dependence of quantum Hall states exhibits characteristic behaviours: the width of the ρ_{xy} plateaus and the minima in ρ_{xx} decrease as temperature increases. The resistance minima in ρ_{xx} for both integer and fractional states follow an "activated behaviour" over a wide temperature range, described by the Arrhenius law:

$$\sigma_{xx} = \sigma_0 \cdot e^{-\frac{\Delta}{2k_B T}} \quad \text{or} \quad \rho_{xx} = \rho_0 \cdot e^{-\frac{\Delta}{2k_B T}},$$

where Δ represents the activation energy from the Fermi energy to the nearest unoccupied extended state, k_B is Boltzmann's constant, T is the temperature, and the factor of 2 comes from the law of mass action.

Power law: At lower temperatures, resistivity no longer follows the Arrhenius law but instead has a power law dependence as shown in literature[6]

$$\rho_{xx} = aT^{\gamma}.$$

As a result, in a log-log plot, as displayed in the right column of Sample S4 and S5 in Figure S11, the power law appears as a linear function. The factor γ defines the exponent with which ρ_{xx} increases with temperature[6].

C. Measurement of the g-factor on resonator S4

When measuring the longitudinal transport on both sides of the S4 Hall bar (see Figure S5), analogously to the data presented in the main text, we observe the lifting of the minima and a worse quantized plateau in the transverse direction with decreasing distance between the resonator plane and the Hall bar sample. Again, we can already

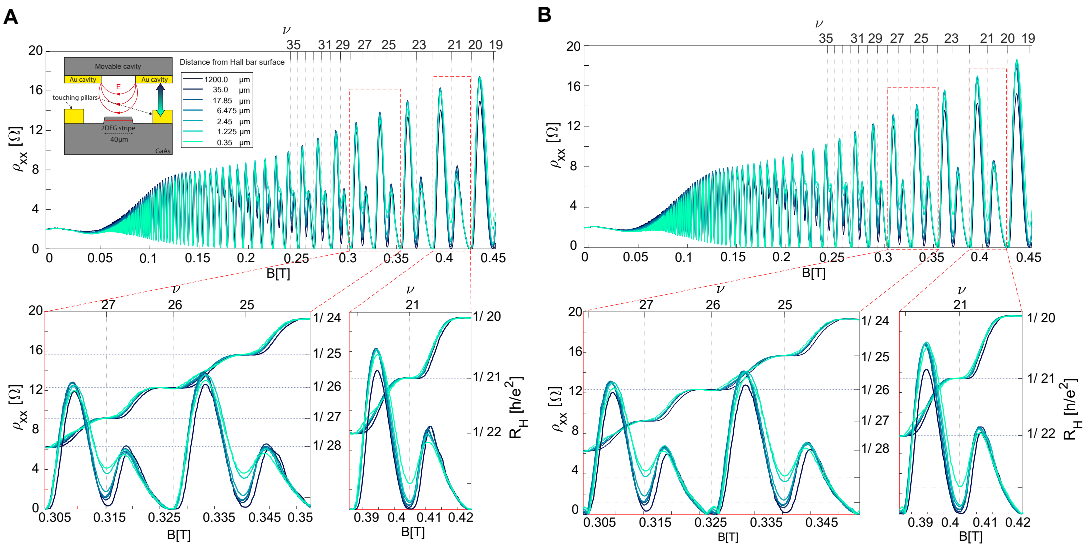

FIG. S5. **S4 data (A)** Data shown for the sample S4 (side 1 - Vxx1) taken at various distances between the cavity and Hall bar. Top: Focus on low field, showing the absence of density and zero-field mobility drifts. Bottom: Zoom in to filling factors 27 to 25 and 21. **(B)** Data shown for sample S4 but on the opposite side (Vxx2) of the Hall bar.

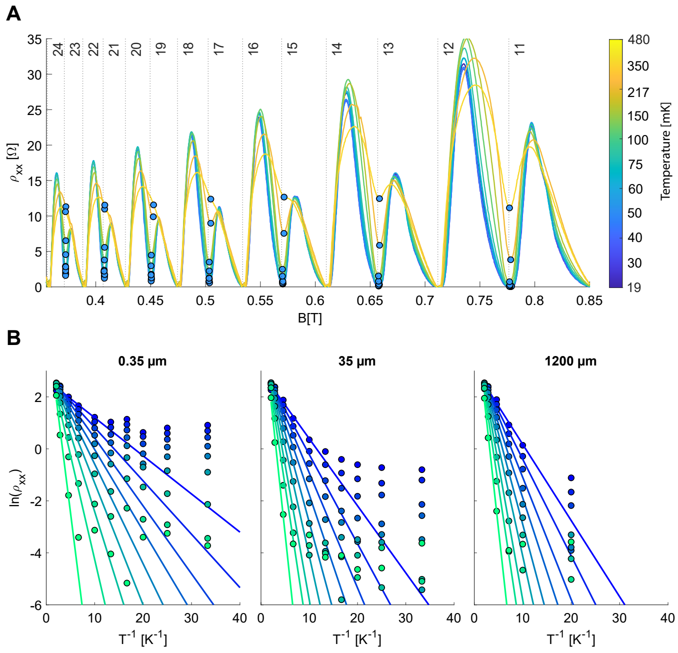

FIG. S6. **Temperature activation and g-factor for sample S4 (A)** Longitudinal resistance measured at multiple temperatures ranging between 19-480 mK. The blue dots mark the position at which the value for the resistivity was extracted. **(B)** Arrhenius plots for the temperature sweeps taken at the different distances 0.35 μm, 35 μm and 1200 μm between the resonator plane and the Hall bar.

observe a hint of the reduction in the Zeeman split states as the peaks between the odd integer filling factors tend to move closer together.

In Figure S6B, the Arrhenius plots on sample S4 (side Vxx2) for the integer filling factors show data points from the longitudinal minima in the resistance plotted on a logarithmic scale as a function of $1/T$. The extracted activation energies for both sides Vxx1 and Vxx2 of sample S4 can be found in Figure S7. The error on the activation energies is the standard deviation of the fit displayed in Figure S6.

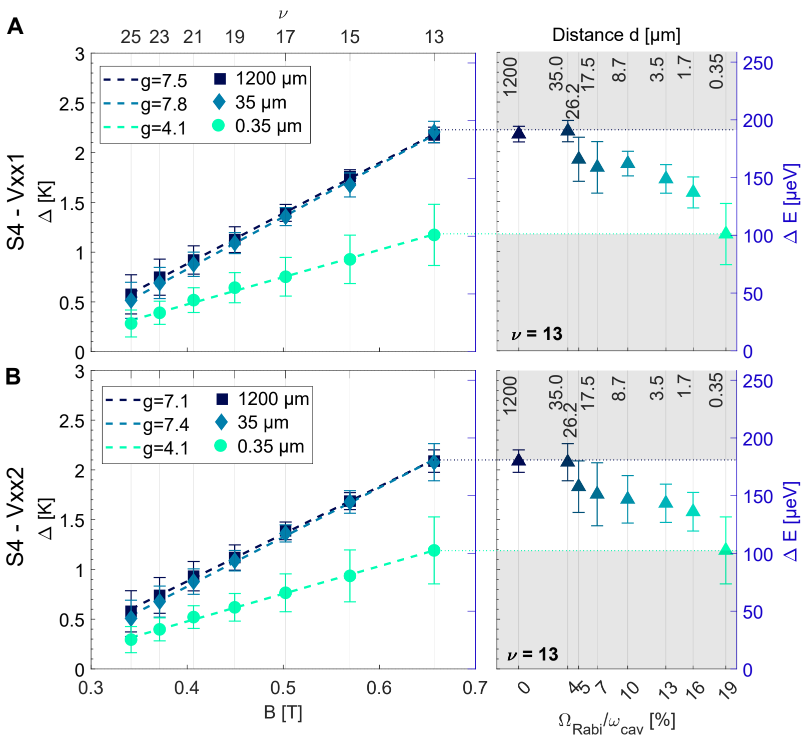

FIG. S7. **(A)** Sample S4, measurements on side Vxx1. Left: Extracted activation energies for odd filling factors 25 to 13 as a function of the magnetic field. Dashed lines indicated the fit of the effective g-factor. Right: Evolution of the activation energy of filling factor 13 as a function of distance. **(B)** Same for sample S4, measured on side Vxx2.

D. Fractional states

1. *Sample S4 - combining the data of both sides Vxx1 and Vxx2*

For the traces taken on sample S4, we combine the longitudinal data taken on the sides Vxx1 and Vxx2 when sweeping the field from 0 T to 11 T to incorporate possible density gradients within the sample[7] into one data set. We merge the two longitudinal resistance traces R_{xx2} and R_{xx1} in the following manner:

$$\tilde{\rho}_{xx\uparrow} = \frac{W}{L} \frac{R_{xx2} - R_{xx1}}{\log(R_{xx2}/R_{xx1})}$$

where W is the width and L is the length of the Hall bar (i.e. the distance between the longitudinal probes). This combined trace ρ_{xx} will be referred to as "both sides" and carries the information of both Vxx1 and Vxx2.

2. *S4 and S5: evaluation of the cavity-driven improvement of the quantization*

The temperature dependence of the fractional states goes over three regimes making the evaluation of the activation temperature challenging. Regardless of the model, we show that there is a *consistent overall improvement of the quantization at low temperatures*. Indeed, in Figure S8, we show the difference between $\rho_{\text{min:c}}$ taken when the cavity is close (0.35 μm - 'c') and when the cavity is 35 μm (far - 'f') or 1200 μm (very far - 'vf') away from the Hall bar as a function of the temperature, that is

$$\Delta\rho_{xx} = \rho_{\text{min:f/vf}} - \rho_{\text{min:c}}.$$

For low temperatures, the sign of $\Delta\rho_{xx}$ for both samples S4 (Fig. S8, left) and S5 (Fig. S8, right) is *almost always* positive: this indicates that the cavity *lowers* the resistance with respect to the situation when the resonator is further away, and the system is less coupled. At higher temperatures, the effect of the cavity on $\Delta\rho_{xx}$ diminishes: in this regime, the resistance is dominated by temperature-induced scattering.

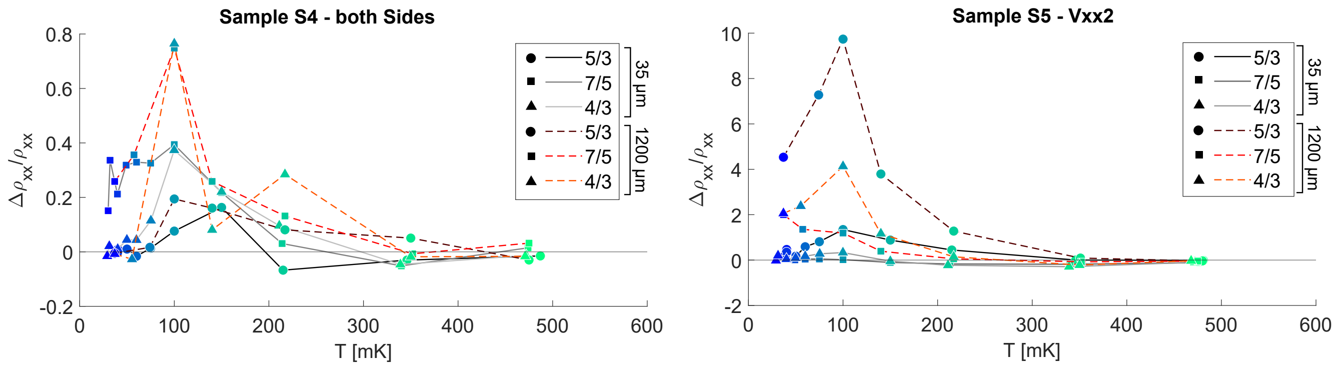

FIG. S8. **Left:** Sample S4 both sides. $\Delta\rho_{xx}/\rho_{xx}$ as a function of the temperature for the fractions 5/3, 7/5 and 4/3. Solid grey lines indicate the difference in $\rho_{\min}$ between the traces taken when the cavity is 35 µm away and when the cavity is 0.35 µm close referenced to when the cavity is 0.35 µm close. The dashed red traces indicate the difference in $\rho_{\min}$ between the traces taken when the cavity is 1200 µm away and when the cavity is 0.35 µm close referenced to when the cavity is 0.35 µm close. **Right:** Same plot of $\Delta\rho_{xx}$ for sample S5 on side Vxx2.

3. *Magnetotransport curves for different distances as a function of temperature for both Hall bars S4 and S5*

Shown in Fig. S9 are the longitudinal resistance measurements as a function of magnetic field for increasing temperatures in the magnetic field range where the fractions 5/3, 7/5 and 4/3 appear. The data are taken for three different distances between the resonator and the Hall bar and for both Hall bar S4 and S5. Indicated are also the location of the minima that are then used for the Arrhenius plots of the resistance. In this temperature analysis, we recorded 11 temperature points for distances of $d = 0.35$ µm and $d = 35$ µm, and 7 temperature points for $d = 1200$ µm, as shown by the colour bar in Figure S9. For the analysis of the fractional states, the exact temperature was determined for each specific magnetic field, as heating effects due to eddy currents occur below $T < 50$ mK. These heating effects are negligible at lower magnetic fields ($B < 1$ T), meaning no temperature error was considered in the analysis of the odd integer filling factors.

4. *Determination of the gap for the fractional states*

The evaluation of the fractional gap is complicated by a number of factors. First, the range of temperature over which the resistance minima do exhibit a clean activated behavior is limited on the high temperature side by the disappearance of the fractional state and towards the low temperature side by the onset of the power law behavior.

Shown in Fig. S10 are the points for Hall bar S5 as a function of inverse temperature, with the range indicated where the linear fits were performed. The data that shows both S4 and S5 with both Arrhenius and power law fits is described in Fig. S11.

Selecting the appropriate temperature range that exhibits a linear dependence on $1/T$ is crucial to obtaining reasonable results from the fit. At high temperatures, additional states beyond the next higher level can become excited, leading to deviations from the expected linear behaviour, which is why in some plots of Figure S10 and S11 points at high temperatures are excluded as they do not follow the linear trend (red crosses).

Similarly, points at very low temperatures are excluded from the fit because the fractional quantisation accuracy becomes limited by non-activated processes such as disorder, causing the resistance minima to saturate, and are therefore excluded from the fit (red crosses). In the lower temperature regime we can exploit the power law dependence of the resistance minima as a function of temperature. In Figure S11, the right columns of each sample, S4 and S5, display the fit of the power law dependence in a log-log plot. The left column of each sample displays the Arrhenius plots. In every column (Arrhenius plot and power law dependence), the points $\rho_{\min}$ extracted for the distances $d = 0.35$ µm (light grey), $d = 35$ µm (grey) and $d = 1200$ µm (black) are plotted on top of each other for better comparability. Similarly, points which do not follow a linear trend (in log-log) are excluded from the fit and marked with a cross: the lighter the colour, the smaller the distance between the resonator and the Hall bar (distances are 0.35 µm, 35 µm and 1200 µm).

We note that for the 5/3 filling fraction, the highest temperature point at $T = 480$ mK has a large error due to temperature. As a result, this point is assigned a lower weight in the weighted fit. However, the large uncertainties from this fit are fully accounted for in the final error of the activation energy (see Figure 3D in the main text). The

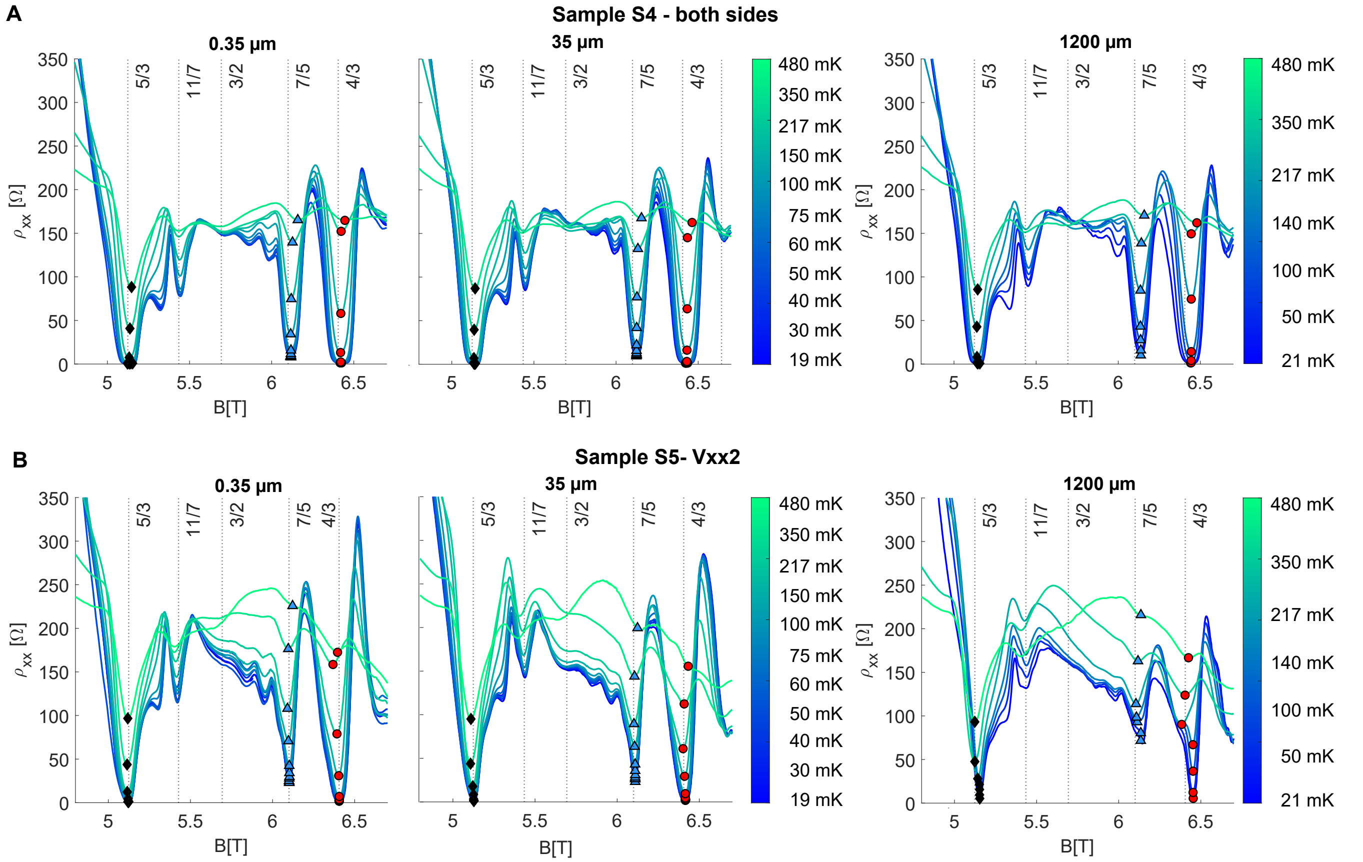

FIG. S9. **(A)** Temperature data taken on the Hall bar sample S4 coupled to the slot antenna and the extracted points ρ_{min} for the fractions 5/3, 7/5 and 4/3. At a distance $d = 0.35$ μm and $d = 35$ μm the longitudinal measurements were taken at temperature (measured at 0 T on the mK-plate of the cryostat) at 11 temperature points. At the distance $d = 1200$ μm on the right 7 temperature points were taken. **(B)** Same plots as in **(A)** for Hall bar sample S5 coupled to the CSRR.

error bars correspond to the 95% confidence bounds of the weighted fits displayed in Figure S11.

To enhance the quality of the fits, we performed a weighted fit that takes into account the noise of the trace. To evaluate the latter, we considered the same magnetic field points arising both when ramping the magnetic field up and down as representing the random part of the measurement. Mathematically, we define the mean

$$\rho_{\text{min}} = \frac{\tilde{\rho}_{xx\uparrow} + \tilde{\rho}_{xx\downarrow}}{2}.$$

while the difference between $\rho_{xx\uparrow}$ and $\rho_{xx\downarrow}$ provides us with an error of the measurement itself

$$\text{var}(\rho_{\text{min}}) = \frac{(\tilde{\rho}_{xx\uparrow} - \rho_{\text{min}})^2 + (\tilde{\rho}_{xx\downarrow} - \rho_{\text{min}})^2}{2}$$

During the sweep, we monitor the temperature of the millikelvin plate (the coldest point in the cryostat) and extract the temperature corresponding to the magnetic field of the fractional states for both up field and down field ramps. Similarly, we evaluate the error arising from the temperature fluctuations

$$\text{var}(T) = \frac{(T_\uparrow - T_{\text{average}})^2 + (T_\downarrow - T_{\text{average}})^2}{2}.$$

We can combine these two contributions of the error and introduce a weight in the following manner:

$$w = \frac{1}{\text{var}(\rho_{\text{min}}) + \text{var}(T)}.$$

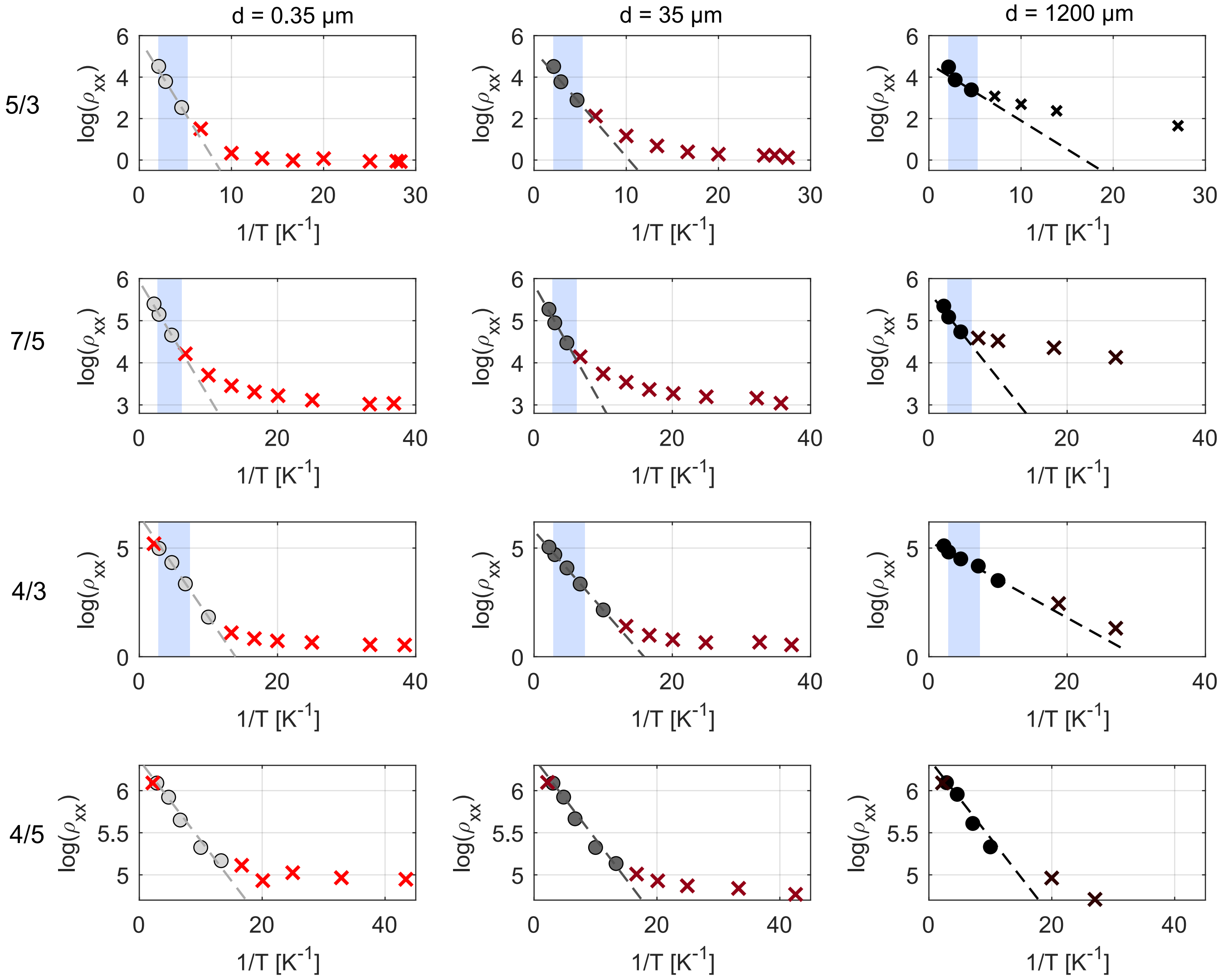

FIG. S10. **Sample S5, side Vxx2**: Arrhenius plots for the fractional states 5/3, 7/5, 4/3 and 4/5 for the distances $d = 0.35$ μm (left column), $d = 35$ μm (middle column) and $d = 1200$ μm (right column). Red crosses indicate points excluded from the weighted fit. The blue shaded area for the fractions 5/3, 7/5 and 4/3 indicates the temperature interval for which the activation temperature in the reference Van der Paw configuration was extracted (see Figure S14).

5. *Results Temperature Analysis for S4 and S5*

Figure S12 displays both the activation energy and the factor γ obtained for the fractions 5/3, 7/5, 4/3 and 4/5 as a function of the coupling and distance for Sample S4 (sample S5 is discussed in the main text, and the analogous results are shown in Fig. 3D,E therein). In a broad picture, the same qualitative behavior is observed as for the sample S5, although the magnitude of the effect is significantly smaller. We see that there is a slight increase in the activation energy (Fig. S12, left) with larger light-matter coupling for the fractions 5/3, 7/5 and 4/3, a trend that is reproduced (Fig. S12, right) in the factor γ obtained from the power law. No such trend is observed for the fraction 4/5, which, as already mentioned in the maint text, we attribute to the fact that the fraction 4/5 is part of another family of fractions. The error bars correspond to the 95% confidence bounds of the fits displayed in Figure S11. Although we do not yet have an understanding of the physics which determines the variation in the value of the γ exponent, its significant change nonetheless visualises and underlines the effect of the cavity on the fractional states.

In general, the size of the fractional gaps as measured in transport do not only depend on the strength of the Haldane potentials but also on experimental details such as the precise disorder in the center of the Hall bar as well as in the contact leads. We believe this effect is especially strong in our samples, in which the current-carrying central section, 40 μm wide as it needs to fit in the gap of the CSRR resonator, is narrower than what is recommended in the

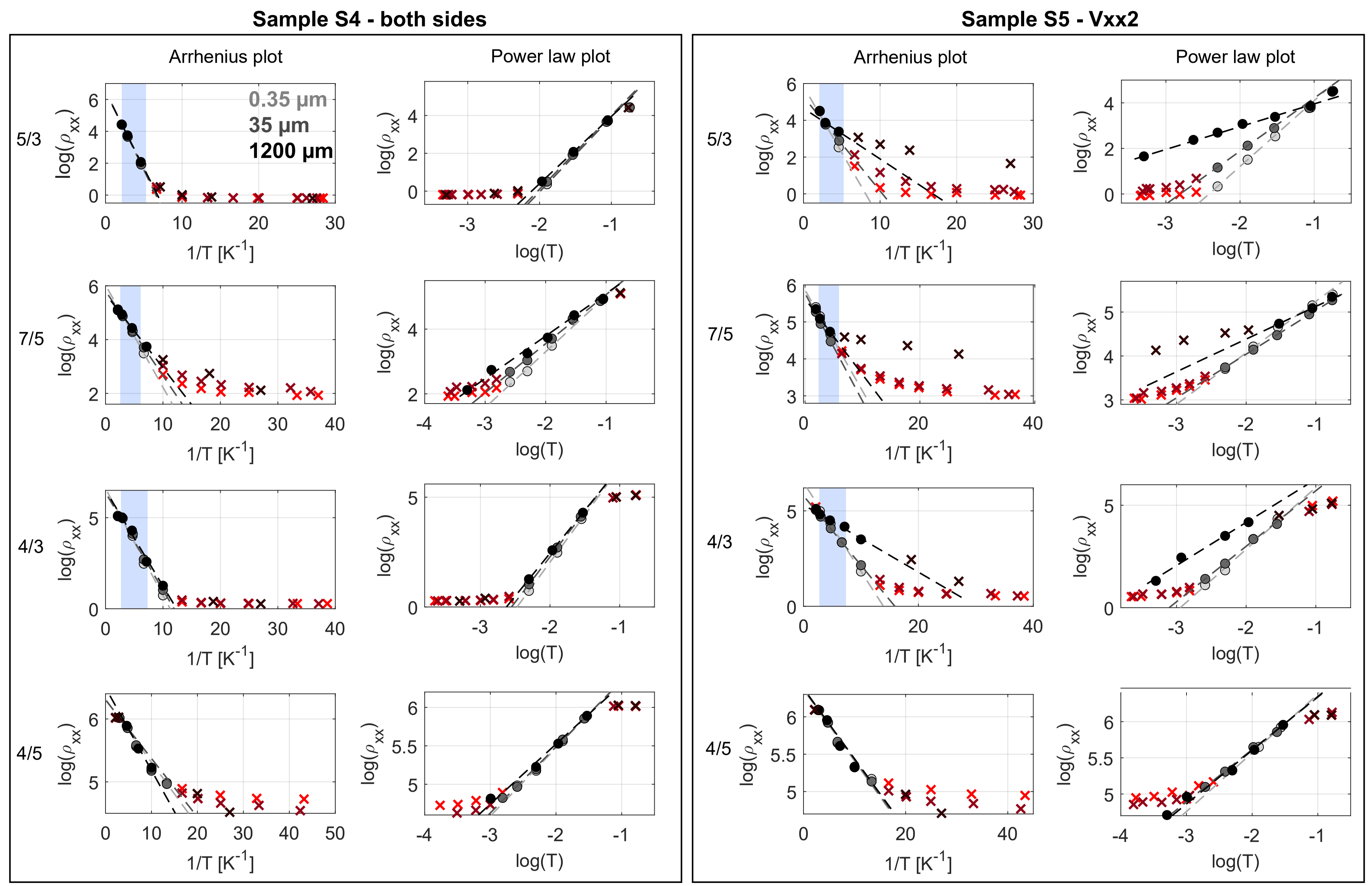

FIG. S11. **Left Box**: Sample S4 both sides. in the left column the Arrhenius plots for the fractional states 5/3, 7/5, 4/3 and 4/5 for the distances $d = 0.35$ μm (light grey), $d = 35$ μm (grey) and $d = 1200$ μm (black) are shown. The crosses indicate the points that were excluded from the fit of the activation and power law, where light red crosses correspond to $d = 0.35$ μm, red to $d = 35$ μm and dark red to $d = 1200$ μm. The blue shaded area indicates the temperature interval for which the activation temperature in the reference Van der Paw configuration was extracted (see Figure S14). Fits are indicated with dashed lines. **Right Box** Same plots for sample S5, side Vxx2. The Arrhenius plot data are the same one presented, separately for each resonator-Hall bar distance, in Fig. S10.

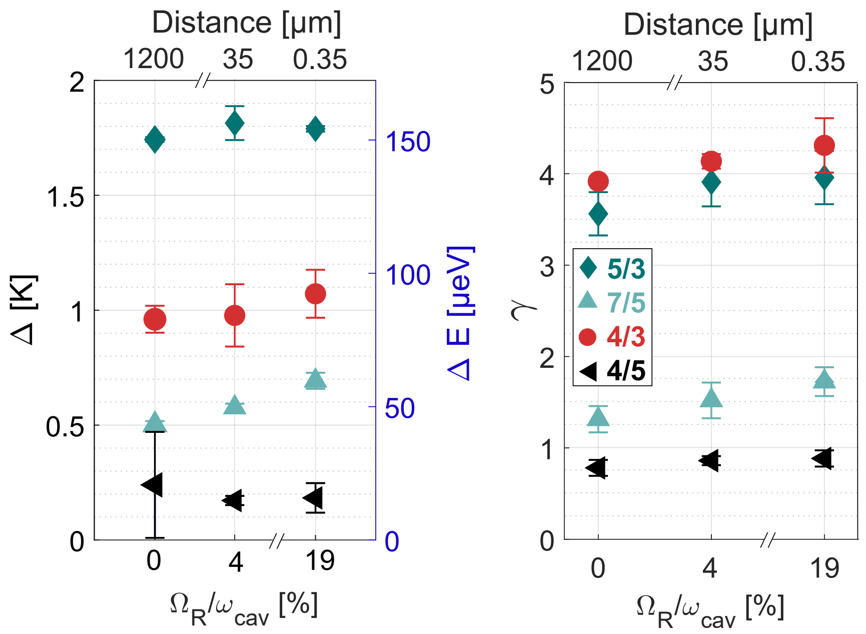

FIG. S12. **Sample S4 results. Left:** Activation energy for the fractions 5/3, 7/5, 4/3 and 4/5 as a function of the coupling (bottom axis) and distance between the Hall bar and the resonator (top axis). **Right:** γ exponent from the power law dependence for the fractions 5/3, 7/5, 4/3 and 4/5 as a function of the coupling (bottom axis) and distance between the Hall bar and the resonator (top axis).

literature for the observation of fractional states. As a result, we attribute the difference between the gaps measured for the two Hall bar S4 and S5 to this different impurity distribution. We believe a similar mechanism is the reason for the lack of quantization improvement of the fractional quantum Hall state activation energies in the measurements performed using our previous approach, i.e. where the resonator was evaporated directly on the surface of the two dimensional electron gas[5]. In that case, the edges of the resonator metallization plane added an additional scattering in the voltage probes. Another difference was the slightly lower mobility of the sample and, finally, unlike our present experiment, the fractional gaps could not be compared *in the exact same Hall bar* with and without cavity.

6. *Measurement of the fractional gaps with van der Pauw's method and comparison with literature data*

In this section we present additional data on fractional gaps measured via the van der Pauw method[8] on the same heterostructure out of which the samples presented in the main text have been processed, and we compare our measured gaps with results from the literature.

In Figure S13 we report the longitudinal resistance as a function of magnetic field, measured at different temperatures between 26 and 1200 mK in a dilution refrigerator, between filling factors 1 and 2. The data have been measured via the van der Pauw method on a $4 \times 4\,\mathrm{mm}^2$ chip with annealed indium droplet contacts at its corners and at the middle of the sides, cleaved from the same D151202B-heterostructure wafer which has been employed to process the samples presented in the main text. Being it a large sample, it shows wide and robust zero-resistance states at fractional fillings 5/3, 8/5, 7/5, and 4/3. The fact that these plateaus are more robust with respect to the ones measured in the geometry presented in the main text—which features a 40 µm-wide Hall bar, with down to 10 µm-wide and about 2 mm-long voltage leads, a design constrained by our need to place it below the movable resonator and within the 50 µm-wide resonator gap—is to be expected, since in wide samples density inhomogeneities are averaged out, and counter-propagating edge states are separated by a broader incompressible bulk.

In Figure S14 we report the resistance minima as a function of inverse temperature for the fractional fillings mentioned above, along with the fitting to Arrhenius's law $R_{xx}^{\min} \propto \exp(-\Delta T/2T)$, from which the energy gaps ΔT can be obtained. These amount to about twice the ones measured in the samples presented in the main text, quantifying the higher robustness of fractional states in wide samples. We observe in particular that the range of linearity of the logarithm of the resistance minima matches the inverse-temperature range over which the same fitting procedure was employed to obtain the fractional gaps in the samples presented in the main text, and discussed in Secs. III D 4, and

III D 5. Hence, these data support the validity of the temperature activation study in obtaining the magnitude of the transport gap. Moreover, they further corroborate the analysis presented in the main text, which relies on the self-referencing scheme enabled by the movable resonator to study the influence of cavity vacuum fluctuations on the magnitude of the fractional gaps.

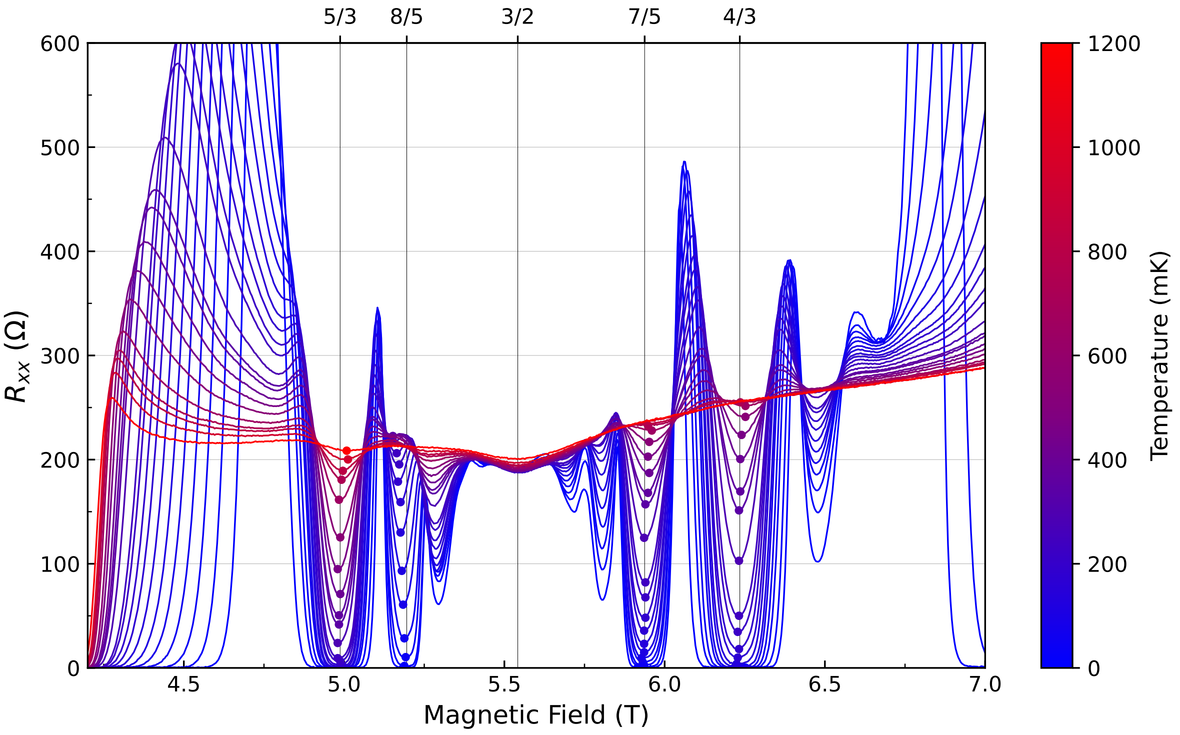

FIG. S13. Longitudinal resistance as a function of magnetic field between filling factors 1 and 2, as measured with van der Pauw's method on the same heterostructure D151202B employed for the samples presented in the main text. Data measured at different temperatures are displayed with different colors, according to the colorbar on the right. The fractional filling factors from which the energy gap is investigated are indicated on the top axis. Minima of the resistance at these fillings are indicated with circle markers, with color according to the temperature.

In Figure S15 we compare the results of the present work with fractional gaps reported in the literature[9–20], as a function of sample mobility. We notice how the gaps measured with van der Pauw's method (blue triangles) follow the trend suggested by previous data, i.e. an increase of the gap with increasing mobility. As already discussed, the gaps measured on the narrow samples presented in the main text are about half the values of the ones measured on a wide sample. However, we can clearly distinguish the fractional gap difference between the case in which the 2DEG is coupled to the vacuum fluctuations (resonator close, red square markers) and the case in which the 2DEG is uncoupled (resonator 1200 µm-far, black square markers).

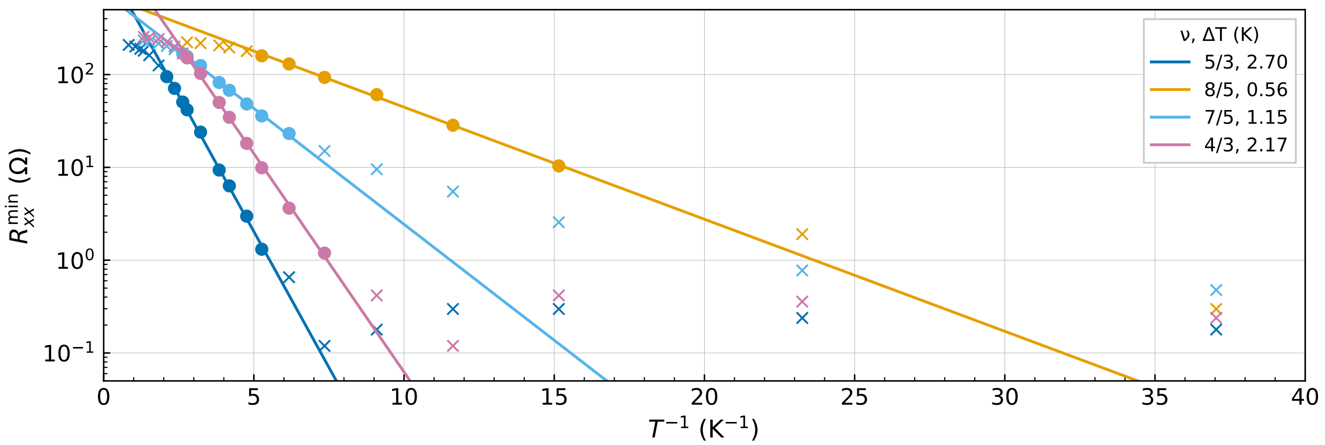

FIG. S14. Minima of the longitudinal resistance (in log scale) as a function of inverse temperature, as obtained from the circle markers in Fig. S13, for the different fractional fillings (indicated in the legend). The data are fitted to the Arrhenius law $R_{xx}^{\min} \propto \exp(-\Delta T/2T)$, which allows to retrieve the fractional gap ΔT, reported in K in the legend, next to the corresponding fractional filling. Circle markers indicate the data points which have been employed in the fitting procedure, since they display a linear dependence (in log scale), while data excluded from the fitting are indicated with cross markers.

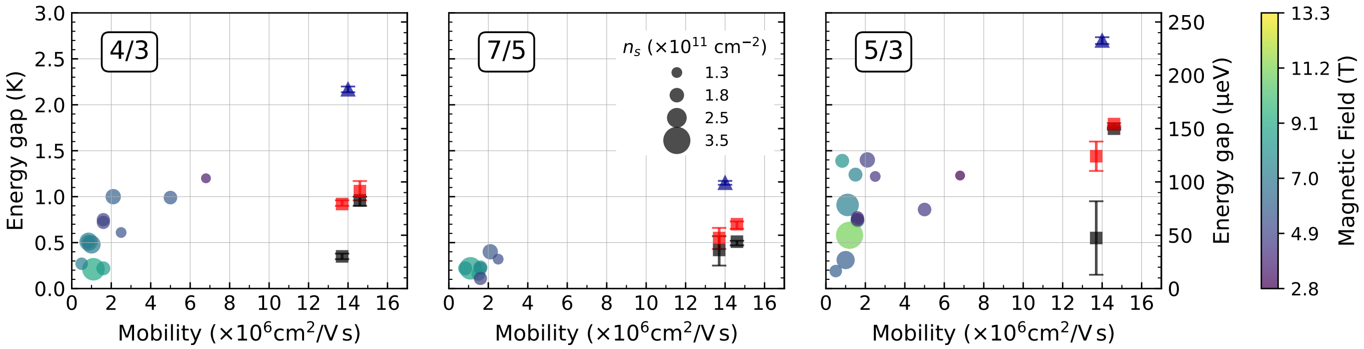

FIG. S15. Energy gaps as a function of mobility for 4/3, 7/5, and 5/3 fractional fillings, comparing the results of the present work (black and red squares), with the measurement empolying the van der Pauw method (blue triangles), and with data from Refs.[9–20] (colored circles). The size of the circle markers indicates the sheet density of the sample on which the gap was measured (according to the legend in the middle panel), while their color indicates the magnetic field at which the corresponding fractional filling is reached (according to the colorbar on the right). The black squares refer to the case in which the resonator plane is 1200 µm far from the Hall bar, while the red squares to the case in which the resonator plane is close to the 2DEG (0.35 µm distance). The gap measured via the van der Pauw method (blue triangles) are almost twice in magnitude with respect to the ones measured in the 40 µm-wide Hall bar geometry.

IV. HAMILTONIAN FORMALISM

A. Hamiltonian of the 2DEG in a single mode cavity

The light-matter interaction Hamiltonian is obtained as follows. We consider $N_{\rm el}$ two-dimensional electrons with effective mass $m_\star$ subject to a perpendicular magnetic field $\mathbf{B} = B\mathbf{e}_z$ and coupled to the quantum field of a single cavity mode. The electromagnetic vector potential seen by the i-th electron is the sum of two contributions: $\mathbf{A_0}(\hat{\mathbf{r}}_i)$ associated with the static magnetic field $\mathbf{B}$ and $\hat{\mathbf{A}}^{\rm cav}(\hat{\mathbf{r}}_i)$ to the cavity field. $\hat{\mathbf{A}}^{\rm cav}(\hat{\mathbf{r}})$ is given in the main text, while in the symmetric gauge $\mathbf{A_0}(\hat{\mathbf{r}}) = \frac{B}{2}(\hat{x}\mathbf{e} - \hat{y}\mathbf{e}_x)$. Including the Coulomb electron-electron repulsive potential $\hat{v}_{ij}$, the corresponding cavity quantum electrodynamical (QED) Hamiltonian reads:

$$\hat{H} = \hbar\omega_{\rm cav}\hat{a}^\dagger\hat{a} + \sum_{i<j}\hat{v}_{ij} + \frac{1}{2m_\star}\sum_{i=1}^{N_{\rm el}}\left(\hat{\mathbf{p}}_i + e\hat{\mathbf{A}}_i\right)^2 . \tag{S2}$$

The operator $\hat{\mathbf{p}}_i$ is the momentum of the i'th electron, $m_\star$ the effective electron mass and $-e$ its charge. Expanding the square in Eq. (S2), the interaction has a paramagnetic and a diamagnetic contribution. The next step of our procedure is to project onto the considered Landau level. The paramagnetic contribution has no intraband contribution. Note that this is not the case when the gradient of the field is not constant.

By introducing fermionic operators for the electrons and using the basis of electronic single-particle states labeled by the angular momentum m, we get $\hat{\mathcal{H}} = \hat{\mathcal{H}}_{\rm cav} + \hat{\mathcal{H}}_{\rm coulomb} + \hat{\mathcal{H}}_{\rm dia}$, where $\hat{\mathcal{H}}_{\rm cav} = \hbar\tilde{\omega}_{\rm cav}\hat{\alpha}^\dagger\hat{\alpha}$, $\hat{\mathcal{H}}_{\rm coulomb} = \frac{1}{2}\sum_{mnpq}\langle m,n|\hat{v}_{12}|q,p\rangle\hat{c}^\dagger_m\hat{c}^\dagger_n\hat{c}_p\hat{c}_q$ and $\hat{\mathcal{H}}_{\rm dia} = \left(\hat{\alpha}^\dagger + \hat{\alpha}\right)^2\hat{\mathcal{H}}^{\rm el}_{\rm dia}$ with

$$\begin{aligned}\hat{\mathcal{H}}^{\rm el}_{\rm dia} &= \tilde{\mathcal{D}}\sum_m\left(\sqrt{m+1}\hat{c}^\dagger_{m+1}\hat{c}_m + \text{h.c.}\right)\\ &+ \frac{\mathcal{D}}{2}\sum_m\left(\sqrt{m+1}\sqrt{m+2}\hat{c}^\dagger_{m+2}\hat{c}_m + \text{h.c.}\right)\\ &+ \mathcal{D}\sum_m m\,\hat{c}^\dagger_m\hat{c}_m .\end{aligned} \tag{S3}$$

Note that $\tilde{\mathcal{D}} = \left(\frac{\omega_{\rm cav}}{\tilde{\omega}_{\rm cav}}\right)\sqrt{2}A_{\rm vac}\mathcal{G}_{\rm A}\ell\left(\frac{e^2}{2m_\star}\right)$ and $\mathcal{D} = \left(\frac{\omega_{\rm cav}}{\tilde{\omega}_{\rm cav}}\right)(\mathcal{G}_{\rm A}\ell)^2\left(\frac{e^2}{2m_\star}\right)$, where $\ell = \sqrt{\hbar/eB}$ is the magnetic length.

B. Effective Interaction

We construct an effective electronic Hamiltonian by adiabatically eliminating the photonic degrees of freedom using the intermediate Hamiltonian technique[21,22].

Let $\{|\phi_i\rangle\}_i$ be the Fock basis of the free electron states. The effective Hamiltonian reads:

$$\begin{aligned}\hat{\mathcal{H}}_{\rm eff} &= \sum_{a,b}\langle\phi_a|\hat{\mathcal{H}}_{\rm dia}|\phi_b\rangle|\phi_a\rangle\langle\phi_b| + \sum_{a,b,i}\frac{\langle\phi_a|\hat{\mathcal{H}}_{\rm dia}|\phi_i,2_{\rm ph}\rangle\langle\phi_i,2_{\rm ph}|\hat{\mathcal{H}}_{\rm dia}|\phi_a\rangle}{E_{|\phi_a\rangle} - E_{|\phi_i,2_{\rm ph}\rangle}}|\phi_a\rangle\langle\phi_b|\\ &= \hat{\mathcal{H}}^{\rm el}_{\rm dia} - \frac{1}{\hbar\tilde{\omega}_{\rm cav}}\left(\hat{\mathcal{H}}^{\rm el}_{\rm dia}\right)^2 .\end{aligned} \tag{S4}$$

The effective electron-electron interaction, reported in the main text of the manuscript, is given by the squared term $\left(\hat{\mathcal{H}}^{\rm el}_{\rm dia}\right)^2$, which contains terms with four fermion operators. The interaction is attractive due to the minus sign. Note that our cavity-mediated electron-electron interaction would describe processes of the type $\hat{c}^\dagger_{m_1}\hat{c}^\dagger_{m_2}\hat{c}_{m'_1}\hat{c}_{m'_2}$ where either the angular momentum is conserved ($m_1 + m_2 = m'_1 + m'_2$) or changed by two ($m_1 + m_2 = m'_1 + m'_2 \pm 2$). There is also the possibility of a unity change ($m_1 + m_2 = m'_1 + m'_2 \pm 1$), but this corresponds to the $\tilde{\mathcal{D}}$-term that can be neglected with respect to the $\mathcal{D}$-term for a macroscopic number of electrons, when we compute the exchange splitting, or even the fractional gap. In such large particle number limit, where the involved angular momenta are large, we can approximate the interaction as conserving angular momentum. The dominant contribution to the effective attractive long-range interaction is reported in the main text. We compute its related Haldane pseudo-potentials $v^{\rm (cav)}_m$ by taking the expectation value of the interaction Hamiltonian on the two-body states with relative angular momentum m. The result is given in the main text.

V. NEGLIGIBLE ROLE OF ELECTROSTATIC SCREENING ON COULOMB POTENTIAL FROM DISTANT HOVERING RESONATOR

A. Experimental aspects

As in our previous work[5], this experiment has been optimized such as to make electrostatic effects negligible. The heterostructure is informally referred to as "ungateable", because of the specific doping technique used to bring the electrons to the high mobility two-dimensional channel. Indeed, the Si dopants are residing in a very thin GaAs layer surrounded by an AlAs quantum well, so a two-dimensional electron channel is formed at the X point of the band-structure of AlAs. Having a very low mobility, these electrons do not contribute to the transport but do screen external static fields, improving the mobility[23]. In addition, the resonator is electrically grounded to the common ground.

We indeed have not observed any indication of a modification of the electron density inside the sample as the hovering resonator is moved, as would have been apparent by a study of the Shubnikov-de Haas oscillations.

B. Theoretical aspects

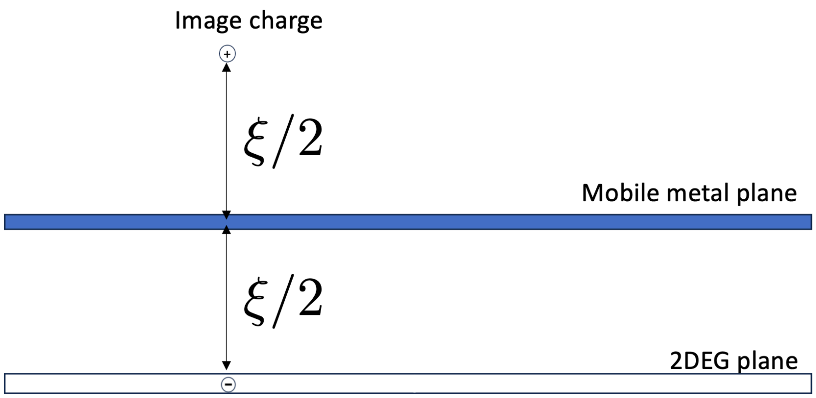

FIG. S16. Sketch depicting the image charge created by a mobile metal plate. ξ is the distance between a charge in the 2D electron gas plane and its opposite image charge.

Here, we present detailed calculations demonstrating that the electrostatic screening effects produced by our metallic hovering resonator are negligible. This is primarily due to the fact that the distance between the 2D electron gas and the mobile metal plane is significantly greater than several tens of times the cyclotron length at the relevant magnetic fields.

Let us calculate the electrostatic screening. A metal plane that is parallel and at a distance $\xi/2$ from the 2D electron gas screens the Coulomb potential of an electron due to an opposite image charge located at the same in-plane position but at an out-of-plane distance ξ from the 2D electron gas (see the sketch in Fig. S16). The screened electrostatic potential reads:

$$V_{\mathrm{scr}}(r;\xi) = \frac{e^2}{4\pi\epsilon_0\epsilon_r}\left[\frac{1}{r} - \frac{1}{\sqrt{r^2+\xi^2}}\right] . \tag{S5}$$

The related Haldane pseudopotentials on a disk geometry are:

$$v_m = \frac{1}{2^{2m+1}\Gamma(m+1)}\int_0^{+\infty} du\, u^{2m+1} V_{\mathrm{scr}}(u\ell;\xi) e^{-\frac{1}{4}u^2} . \tag{S6}$$

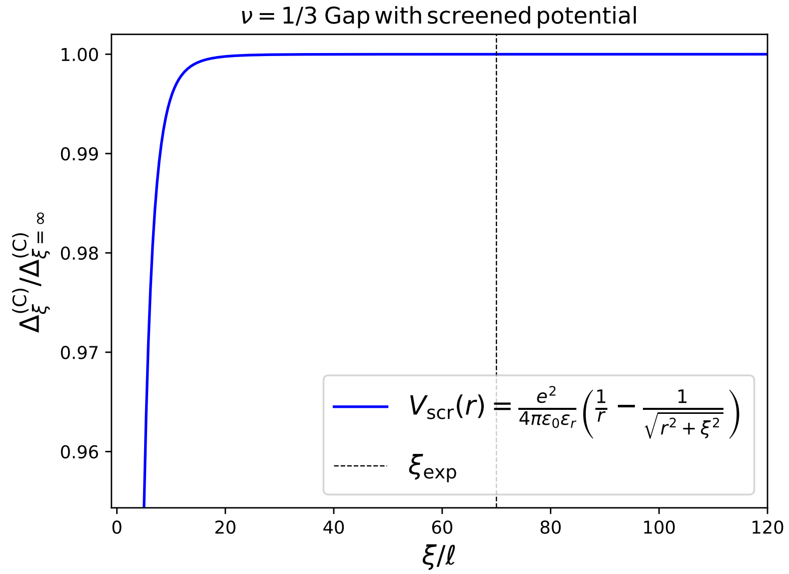

FIG. S17. Evolution of the 1/3 fractional quantum Hall energy gap with a Coulomb potential screened by a metal plane at a distance $\xi/2$. The gap is normalized to the same quantity calculated for the bare Coulomb potential. The distance is expressed in units of the magnetic length ℓ. The result has been obtained by exact diagonalization with $N = 5$ electrons. In the experiments $\xi > 2d = 700$ nm (due to the GaAs buffer on top of the quantum well). For $B = 5$ T, the magnetic length $\ell \simeq 10$ nm. Hence, for our experimental set-up, we have $\xi/\ell > 70$. As shown by the figure, the variation of the fractional quantum Hall gaps is totally negligible at such distances. Note also the tiny decrease of the gap at much shorter distances.

In our experimental configuration, the mobile metal plane is at a distance $\xi/2 > d \geq 350$ nm, d being the distance from the 2DEG to the mobile metal plane, where we have not even included the GaAs buffer (250nm-thick) separating the 2D electron gas from air. In the region of interest, the magnetic field is around 5 T, yielding a magnetic length $\ell \approx 10$ nm. Hence, the ratio $\xi/\ell > 70$. At these distances, the electrostatic screening effects on the fractional quantum Hall phases should be completely negligible. The results of our exact diagonalization results in Figure S17 show exactly that. We report how the 1/3 fractional quantum Hall gap changes as a function of the ratio ξ/ℓ. In particular the gap with the electrostatically screeened potential is normalized to the gap with the bare Coulomb potential. The results show clearly that the variation of the gap for $\xi/\ell = 70$ is totally negligible. Note that moreover at short distances (order of the magnetic length) the fractional quantum Hall gap does not increase, but decreases, hence contrary to the experiments.

Note also that our theoretical calculations agree with the results of a previous theoretical paper[24], which studied the effect of a dielectric in close proximity to a 2D electron gas. They demonstrated that effects occur only when the distance is comparable to the magnetic length. Moreover, the metallic case corresponds to $\alpha = \frac{\epsilon_1-\epsilon_2}{\epsilon_1+\epsilon_2} = -1$ in Ref.[24] (for a metal the absolute value of the dielectric constant $|\epsilon_2| \gg |\epsilon_1|$ where ϵ_1 is the dielectric constant of the 2D material). The results in Ref.[24] indeed show a reduction of the fractional quantum Hall effect rather than an increase for the case $\alpha = -1$.

VI. EXACT DIAGONALIZATION RESULT WITH AN EFFECTIVE CAVITY-MEDIATED POTENTIAL

Here we report exact diagonalization results for a small number of electrons in a disk geometry. We show that adding an attractive cavity-mediated potential to the Coulomb repulsive interaction we can get an enhancement of fractional quantum Hall gaps. Let us consider our cavity-mediated potential

$$\tilde{V}^{(\mathrm{cav})}(r) = \left(-\frac{\mathscr{D}^2}{8\hbar\tilde{\omega}_{\mathrm{cav}}}\right)\left[\frac{1}{16}\left(\frac{r}{\ell}\right)^4 - \left(\frac{r}{\ell}\right)^2 + 2\right] e^{-\frac{1}{2}\left(\frac{r}{L_{\mathrm{c}}}\right)^2}, \tag{S7}$$

where we have introduced a gaussian cutoff characterized by the cutoff length L_{c} . Note that our potential has been calculated considering a constant spatial gradient of the vacuum electric field and would normally diverge when the distance r goes to infinity. This is due to the simplifying approximation of a constant gradient. The gaussian cutoff regularizes such divergence.

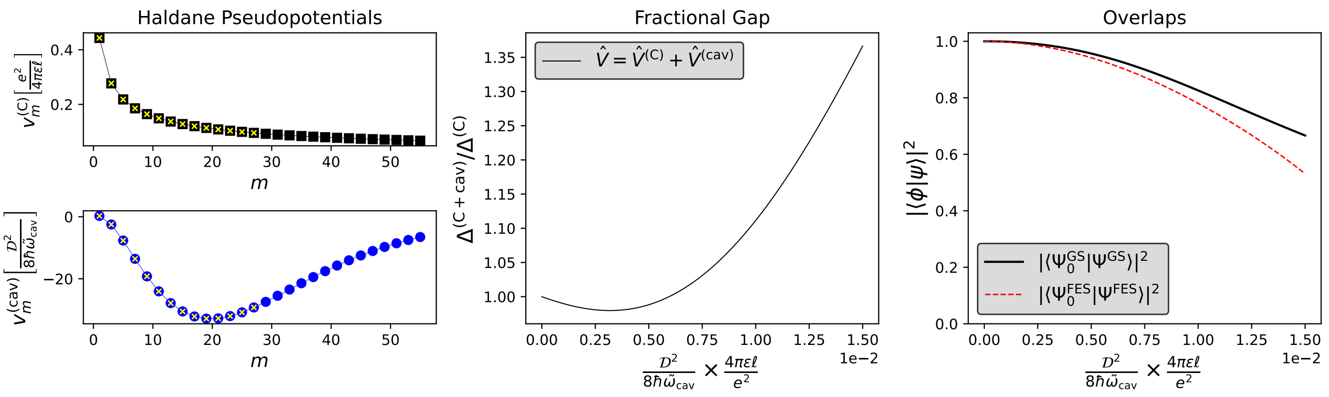

FIG. S18. **Left panels**: the odd Haldane pseudopotential components versus the angular momentum for the repulsive Coulomb interaction (top) and for the attractive cavity-mediated interaction (bottom) with an exponential gaussian cutoff (see text for details). The crosses indicate the angular momenta that count in the case with $N = 5$ electrons. **Central panel**: exact diagonalization results for the fractional quantum Hall gap with $N = 5$ electrons for filling factor equal to $1/3$. The gap is normalized to the gap without the cavity interaction. The horizontal axis displays the amplitude of the attractive potential. **Right panel**: the solid line depicts the overlap between the ground state with the cavity-mediated potential and the ground state with no cavity coupling; the red dashed line represents the overlap for the first excited state.

To perform exact diagonalization, it is convenient to get the Haldane pseudopotential components for such potential:

$$\tilde{v}_m^{(\text{cav})} = \frac{1}{2^{2m+1}\Gamma(m+1)} \int_0^{+\infty} du\; u^{2m+1} \tilde{V}^{(\text{cav})}(u\ell) e^{-\frac{1}{4}u^2}. \tag{S8}$$

After some algebra, the Haldane components read

$$\tilde{v}_m^{(\text{cav})} = \left(-\frac{\mathcal{D}^2}{8\hbar\tilde{\omega}_{\text{cav}}}\right) e^{-\lambda_c m} \left[e^{-3\lambda_c}(m^2+3m+2) - 4e^{-2\lambda_c}(m+1) + 2e^{-\lambda_c}\right], \tag{S9}$$

where $m_c = (L_c/\sqrt{2}\ell)^2$ and $\lambda_c = \log\left(\frac{m_c+1}{m_c}\right)$. Note that the gaussian cutoff for the potential in real space corresponds to an exponential cutoff of the Haldane components at large values of the angular momentum m.

Figure S18 reports exact diagonalization results for 5 electrons. The left panels show the Haldane components for the Coulomb repulsive interaction (top) and the cavity-mediated attractive interaction (bottom). The central panel shows results for the $1/3$ fractional quantum Hall gap versus the amplitude of the cavity-mediated interaction. The gap is normalized to the value of the gap with no cavity coupling. The amplitude of the cavity-mediated potential is expressed in Coulomb units. Remarkably we do observe an enhancement of the gap. Note that for these calculations with only 5 electrons, we use artificially large values of the cavity-mediated potential amplitude, because we cannot use exact diagonalization for the very large number of electrons that are present in the experiments. In particular, we cannot use it to explore collective effects due to the long-range potential. The solid line in the right panel of Figure S18 reports the overlap between the ground state modified by the cavity vacuum fields with the ground state without. It is apparent that there is a smooth and continuous decrease of such overlap. A gap increase by approximately 35% (central panel) corresponds to an overlap of about 70%. The dashed line displays the overlap for the first excited state, which exhibits a similar behavior.

VII. MAGNETO-ROTON THEORETICAL RESULTS FOR THE FRACTIONAL QUANTUM HALL GAPS

Here, we apply the magneto-roton theory of Girvin, MacDonald, and Platzman[25] to estimate the variation in fractional quantum Hall gaps produced by the cavity vacuum fields. This theory is based on a successful ansatz, similar to the one introduced by Feynman to describe excitations in superfluid helium, but restricted to the lowest Landau level. The only inputs needed are $V(q)$, the Fourier transform of the electron-electron potential in momentum space, and $\bar{s}(k)$, the static structure function. The static structure function is related to the Fourier transform of

the density-density correlation function (with the density operator projected onto the lowest Landau level) for the considered fractional quantum Hall ground state.

The Fourier transform of the real-space potential in Eq. (S7) reads:

$$V^{(\mathrm{cav})}(q) = -\left(\frac{\mathcal{D}^2}{8\hbar\tilde{\omega}_{\mathrm{cav}}}\right)(2\pi L^2)\left\{\frac{L^4}{16\ell^4}(qL)^4 + \left[-\frac{L^4}{2\ell^4}+\frac{L^2}{\ell^2}\right](qL)^2 + \left[\frac{L^4}{2\ell^4} - 2\frac{L^2}{\ell^2} + 2\right]\right\} e^{-\frac{1}{2}(Lq)^2} . \tag{S10}$$

The static structure factor for the lowest Landau level reads[25]

$$\bar{s}(\boldsymbol{k}) = s(\boldsymbol{k}) - (1 - e^{-\frac{1}{2}(\ell|\boldsymbol{k}|)^2}) , \tag{S11}$$

where

$$s(\boldsymbol{k}) = \rho(2\pi)^2\delta^{(2)}(\boldsymbol{k}) + 1 - \rho\int d^2r e^{-\mathrm{i}\boldsymbol{k}\cdot\boldsymbol{r}}[g(\boldsymbol{r}) - 1] \tag{S12}$$

with ρ being the density of the 2D electron gas and $g(\boldsymbol{r})$ is the density-density correlation function of the ground state.

With our long-range potential we need to regularize the theory by introducing a cutoff at long distances. For finite-size samples, we also have to regularize the Dirac delta distribution in the static function. For simplicity, we will consider the following representation of the delta function:

$$\delta_L^{(2)}(\boldsymbol{k}) = \frac{L^2}{\pi}\mathbf{1}_{|\boldsymbol{k}|<\frac{1}{L}} . \tag{S13}$$

Note that we have $\int d^2q\delta_L^{(2)}(\boldsymbol{k}) = 1$ and $\lim_{L\to+\infty}\delta_L^{(2)}(\boldsymbol{k}) = 0$ for all $\boldsymbol{k} \neq \boldsymbol{0}$.

The expression of the magnetoroton gap is:

$$\Delta = \min_k \frac{\bar{f}(k)}{\bar{s}(k)} , \tag{S14}$$

where

$$\bar{f}(k) = \int \frac{d^2q}{(2\pi)^2} V(q)\left\{1 - \cos\left[\ell^2\left(\boldsymbol{k}\times\boldsymbol{q}\right)_z\right]\right\} \times \times\left[\bar{s}(\boldsymbol{k}+\boldsymbol{q})e^{\ell^2\boldsymbol{k}\cdot\boldsymbol{q}} - \bar{s}(\boldsymbol{q})e^{-\frac{1}{2}(\ell\boldsymbol{k})^2}\right] \tag{S15}$$

is the lowest Landau level projected oscillator strength. Hence the variation of the gap due to the cavity is given by the expression:

$$\Delta^{(\mathrm{cav+C})} - \Delta^{(\mathrm{C})} = \min_k \frac{\bar{f}^{(\mathrm{cav})}(k)}{\bar{s}(k)} , \tag{S16}$$

where we have assumed that the ground state remains unperturbed by the cavity field, only the excited state being changed. This means that the static structure factor remains the same for the bare and cavity cases. In the limit $L \gg l$ we have

$$\begin{aligned} \bar{f}_L^{(\mathrm{cav})}(k) &\simeq \left(\frac{L}{\ell}\right)^4\left(\frac{\mathcal{D}^2}{8\hbar\tilde{\omega}_{\mathrm{cav}}}\right)(\ell k)^2 e^{-\frac{1}{2}(\ell k)^2} \times \\ &\times\left(\frac{\nu}{8\pi^2}\right)\left[\int_0^1 d\eta\left(\frac{1}{16}\eta^7 - \frac{1}{2}\eta^5 + \frac{1}{2}\eta^3\right)e^{-\frac{1}{2}\eta^2}\right] \\ &\simeq 0.04\left(\frac{L}{\ell}\right)^4\left(\frac{\mathcal{D}^2}{8\hbar\tilde{\omega}_{\mathrm{cav}}}\right)(\ell k)^2 e^{-\frac{1}{2}(\ell k)^2}\left(\frac{\nu}{8\pi^2}\right) . \end{aligned} \tag{S17}$$

We point out that that the long-range nature of the cavity-mediated interaction is responsible for the extensive scaling of the variation of the excitation gap. Note that the k-dependence of $\bar{f}_L^{(\mathrm{cav})}(k)$ is smooth and does not shift significantly the magneto-roton minimum wavevector k_{min} even when the gap is increased by 50% (see Figure S19).

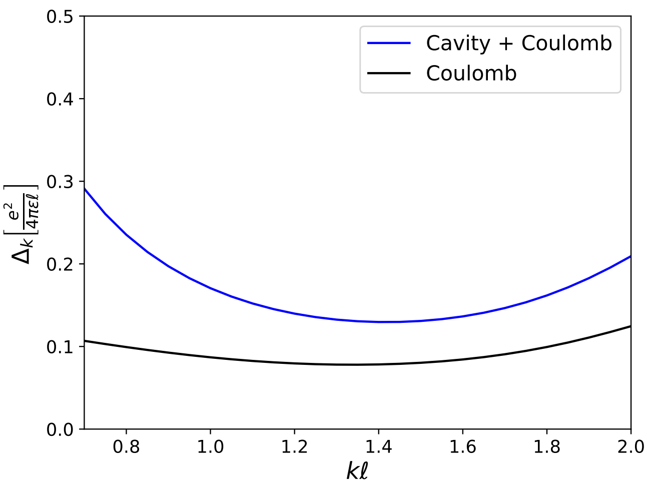

FIG. S19. Magnetoroton energy-momentum dispersion for the 1/3-state without cavity (black line) and with the cavity (blue line). Parameters are the same discussed in the main text.

A more detailed derivation of the theory with additional studies will appear in a forthcoming publication[26].

[1] D. Hagenmüller, S. D. Liberato, and C. Ciuti, Ultrastrong coupling between a cavity resonator and the cyclotron transition of a two-dimensional electron gas in the case of an integer filling factor, Physical Review B **81**, 10.1103/physrevb.81.235303 (2010).
[2] J. J. Hopfield, Theory of the contribution of excitons to the complex dielectric constant of crystals, Phys. Rev. **112**, 1555 (1958).
[3] G. L. Paravicini-Bagliani, F. Appugliese, E. Richter, F. Valmorra, J. Keller, M. Beck, N. Bartolo, C. Rössler, T. Ihn, K. Ensslin, *et al.*, Magneto-transport controlled by landau polariton states, Nature Physics **15**, 186 (2019).
[4] G. L. Paravicini-Bagliani, G. Scalari, F. Valmorra, J. Keller, C. Maissen, M. Beck, and J. Faist, Gate and magnetic field tunable ultrastrong coupling between a magnetoplasmon and the optical mode of an LC cavity, Physical Review B **95**, 10.1103/physrevb.95.205304 (2017).
[5] F. Appugliese, J. Enkner, G. Paravicini-Bagliani, M. Beck, C. Reichl, W. Wegscheider, G. Scalari, C. Ciuti, and J. Faist, Breakdown of topological protection by cavity vacuum fields in the integer quantum Hall effect, Science **375**, 1030 (2022).
[6] J. Matthews and M. Cage, Temperature dependence of the hall and longitudinal resistances in a quantum hall resistance standard, Journal of Research of the National Institute of Standards and Technology **110**, 497 (2005).
[7] R. Ilan, N. Cooper, and A. Stern, Longitudinal resistance of a quantum hall system with a density gradient, Physical Review B—Condensed Matter and Materials Physics **73**, 235333 (2006).
[8] L. van der Pauw, A method of measuring specific resistivity and hall effect of discs of arbitrary shape., Philips Res. Rep. , 1 (1958).
[9] G. Ebert, K. Von Klitzing, J. Maan, G. Remenyi, C. Probst, G. Weimann, and W. Schlapp, Fractional quantum hall effect at filling factors up to ν= 3, Journal of Physics C: Solid State Physics **17**, L775 (1984).
[10] G. Boebinger, A. Chang, H. Stormer, and D. Tsui, Competition between neighboring minima in the fractional quantum hall effect, Physical Review B **32**, 4268 (1985).
[11] G. S. Boebinger, A. Chang, H. Stormer, and D. C. Tsui, Magnetic field dependence of activation energies in the fractional quantum hall effect, Physical review letters **55**, 1606 (1985).
[12] R. Clark, R. Nicholas, A. Usher, C. Foxon, and J. Harris, Odd and even fractionally quantized states in gaas-gaalas heterojunctions, Surface Science **170**, 141 (1986).
[13] R. Willett, H. Stormer, D. Tsui, A. Gossard, and J. English, Quantitative experimental test for the theoretical gap energies in the fractional quantum hall effect, Physical Review B **37**, 8476 (1988).
[14] R. Clark, J. Mallett, S. Haynes, P. Maksym, J. Harris, and C. Foxon, Experimental determination of fractional charge e/q in the fqhe and its application to the destruction of states, in *High Magnetic Fields in Semiconductor Physics II*, edited by L. Gottfried (Springer Series in Solid-State Sciences, 1989) pp. 127, 131.
[15] R. Clark, S. Haynes, A. Suckling, J. Mallett, P. Wright, J. Harris, and C. Foxon, Spin configurations and quasiparticle fractional charge of fractional-quantum-hall-effect ground states in the n= 0 landau level, Physical review letters **62**, 1536 (1989).
[16] A. Sachrajda, R. Boulet, Z. Wasilewski, P. Coleridge, and F. Guillon, Activation measurements of the fractional quantum

hall effect as a function of magnetic field, Solid state communications **74**, 1021 (1990).
[17] N. Morawicz, K. Barnham, C. Zammit, J. Harris, C. Foxon, and P. Kujawinski, Observation of the fractional quantum hall effect under hydrostatic pressure, Physical Review B **41**, 12687 (1990).
[18] N. Morawicz, K. Barnham, A. Briggs, C. Foxon, J. Harris, S. Najda, J. Portal, and M. Williams, Enhancement of the 4/3 fractional quantum hall state with hydrostatic pressure, Semiconductor science and technology **8**, 333 (1993).
[19] R. Du, A. Yeh, H. Stormer, D. Tsui, L. Pfeiffer, and K. West, g factor of composite fermions around ν= 2 from angular-dependent activation-energy measurements, Physical Review B **55**, R7351 (1997).
[20] A. Endo, N. Shibata, and Y. Iye, Collapse of the fractional quantum hall state by a unidirectional periodic potential modulation, Physica E: Low-dimensional Systems and Nanostructures **42**, 1042 (2010).
[21] C. Cohen-Tannoudji, G. Grynberg, and J. Dupont-Roc, *Atom-Photon Interactions: Basic Processes and Applications* (Wiley, New York, 1992).
[22] J.-P. Malrieu, P. Durand, and J.-P. Daudey, Intermediate hamiltonians as a new class of effective hamiltonians, Journal of Physics A: Mathematical and General **18**, 809 (1985).
[23] C. Reichl, J. Chen, S. Baer, C. Rössler, T. Ihn, K. Ensslin, W. Dietsche, and W. Wegscheider, Increasing the $\nu = 5/2$ gap energy: an analysis of MBE growth parameters, New Journal of Physics **16**, 023014 (2014), publisher: IOP Publishing.
[24] Z. Papić, R. Thomale, and D. A. Abanin, Tunable electron interactions and fractional quantum Hall states in graphene, Phys. Rev. Lett. **107**, 176602 (2011).
[25] S. M. Girvin, A. H. MacDonald, and P. M. Platzman, Magneto-roton theory of collective excitations in the fractional quantum Hall effect, Phys. Rev. B **33**, 2481 (1986).
[26] D. Boriçi, N. Regnault, and C. Ciuti, (2025), manuscript in preparation.
